# ﻿An Amazonian hidden gem: a new metallic-colored species of *Ranitomeya* (Anura, Dendrobatidae) from Juruá River basin forests, Amazonas state, Brazil

**DOI:** 10.3897/zookeys.1236.146533

**Published:** 2025-04-25

**Authors:** Alexander Tamanini Mônico, Esteban Diego Koch, Jussara Santos Dayrell, Jiří Moravec, Albertina Pimentel Lima

**Affiliations:** 1 Coordenação de Biodiversidade, Instituto Nacional de Pesquisas da Amazônia, Manaus, Amazonas, Brazil Coordenação de Biodiversidade, Instituto Nacional de Pesquisas da Amazônia Manaus Brazil; 2 Programa de Pós-Graduação em Genética, Conservação e Biologia Evolutiva, Instituto Nacional de Pesquisas da Amazônia, Manaus, Amazonas, Brazil Conservação e Biologia Evolutiva, Instituto Nacional de Pesquisas da Amazônia Manaus Brazil; 3 Department of Zoology, National Museum of the Czech Republic, Cirkusová 1740, 193 00 Prague 9, Czech Republic National Museum of the Czech Republic Prague Czech Republic

**Keywords:** Advertisement call, Amphibia, biodiversity, integrative taxonomy, morphology, phylogeny

## Abstract

The genus *Ranitomeya* has 16 known species, and the last of them was described 13 years ago. The forests of the Juruá River basin are known for their enormous vertebrate diversity, despite being one of the least sampled regions in the entire Amazonia. Our recent expeditions to the region resulted in the discovery of a *Ranitomeya* species with blue-green dorsal stripes and quite peculiar behavior. Here, it is described as a new species using morphological, morphometric, advertisement call, natural history, and genetic data. This new species is strongly nested within the *R.vanzolinii* clade, with interspecific *p*-distances ranging from 2.94 to 3.91%, and it was confirmed in all the delimitation methods used. It differs from its closest relatives mainly by (i) its size (male SVL 15.4–17.7 mm, *n* = 8; female SVL 17.3–18.5 mm, *n* = 5), (ii) its unique color pattern that is metallic pale yellowish green to metallic pale turquoise-green dorsal stripes pattern, limbs metallic chrome with dark carmine spotting), (iii) presence of a conspicuous sulfur yellow spot on the dorsal surface of the thighs, (iv) tadpoles with posterior tooth rows P1 > P2 > P3 in all stages, head translucent brownish and lack of emarginate lateral papillae, and (v) its advertisement call (composed of 21–45 notes, call duration of 647–1,424 ms, note rate of 28–36 notes/s and dominant frequency of 4,996–6,288 Hz).

## ﻿Introduction

The genus *Ranitomeya* Bauer, 1986 currently comprises 16 recognized species (Frost 2025), with the most recent species being described more than 13 years ago (Brown et al. 2011). These species are distributed throughout northern South America, from the Andean foothills to the Amazonian forests (Frost 2025). Among dendrobatid frogs, the genus *Ranitomeya* has posed significant taxonomic challenges because of its high intraspecific morphological variation and mimicry, especially in coloration patterns. These challenges have been compounded by cases where taxonomic studies lacked support from molecular data (Muell et al. 2022).

Currently, *Ranitomeya* is organized into the following five monophyletic species groups (Muell et al. 2022), which were defined by the phylogenetic placement, morphology, mating systems, and vocalization:

**(i) *R.defleri* species group**

*R.defleri* Twomey & Brown, 2009

**(ii) *R.reticulata* species group**

*R.benedicta* Brown, Twomey, Pepper & Sanchez-Rodriguez, 2008

*R.fantastica* (Boulenger, 1884); *R.reticulata* (Boulenger, 1884)

*R.summersi* Brown, Twomey, Pepper & Sanchez-Rodriguez, 2008

*R.uakari* (Brown, Schulte & Summers, 2006); *R.ventrimaculata* (Shreve, 1935)

An undescribed species phylogenetically close to *R.uakarii*

An undescribed species phylogenetically close to *R.benedicta*

**(iii) *R.vanzolinii* species group**

*R.cyanovittata* Pérez-Peña, Chávez, Twomey & Brown, 2010

*R.flavovittata* (Schulte, 1999)

*R.imitator* (Schulte, 1986)

*R.sirensis* (Aichinger, 1991)

*R.vanzolinii* (Myers, 1982)

*R.yavaricola* Pérez-Peña, Chávez, Twomey & Brown, 2010

An undescribed species related to *R.sirensis* from eastern Peru (*R.sirensis* “*biolat*”)

**(iv) *R.variabilis* species group**

*R.amazonica* (Schulte, 1999)

*R.variabilis* (Zimmermann & Zimmermann, 1988)

**(v) *R.toraro* species group**

*R.toraro* Brown, Caldwell, Twomey, Melo-Sampaio & Souza, 2011

In general, numerous studies of the genus have focused on ecological aspects (Lötters et al. 2007), mating systems (Werner et al. 2010; Brown et al. 2011), taxonomy (Brown et al. 2011), biogeography (Muell et al. 2022), and coloration evolution (Vences et al. 2003; Twomey et al. 2023). Despite this, species complexes have been recognized and are yet to be resolved (Brown et al. 2008; Brown et al. 2011; Muell et al. 2022).

The greatest diversity of species is concentrated in eastern-central and north-eastern Peru (Muell et al. 2022), with considerably fewer species along the central Amazonian plain. Meanwhile, despite its vast territorial extension, Brazil has seven species with recorded occurrences (Segalla et al. 2021), and in only two cases their type locality lies on the territory of Brazil (i.e., *R.toraro* and *R.vanzolinii*; Frost 2025). This smaller diversity, aside from being an effect of the species biology, could be caused by a lack of sampling in many areas of the western Brazilian Amazonia.

The forests of the Juruá River basin, a southwestern tributary of the Amazonas River, are known for their enormous vertebrate diversity, despite being one of the least sampled regions in the entire Amazonia (Del-Rio et al. 2021). These knowledge gaps are a result of the exhausting logistics needed to study remote areas, which make it difficult to develop long-term monitoring that allows understanding of diversity patterns (Moraes et al. 2022). For amphibians, most records come from specific inventories, especially from the upper reaches of the basin (e.g., Pantoja and Fraga 2012; Bernarde et al. 2013; Fonseca et al. 2019). Even inventories in areas where *Ranitomeya* is known to occur did not record them, most probably due to their shyness (Brown et al. 2011), which leads to rare encounters. Taxonomically, the region has a huge potential for finding new species, with several candidate lineages already identified (e.g., Moraes et al. 2022; Souza et al. 2023; Lima et al. 2024; Martins et al. 2024). Added to this, it is known that one of the most notable biogeographic patterns for the basin is that species composition appears to be better divided from the lower to upper reaches of the basin than between river margins (Haffer 1997; Matocq et al. 2000; Azevedo-Ramos and Galatti 2002; Tuomisto et al. 2019).

Our recent expeditions to the Eiru and Juruá rivers resulted in the discovery of a new species of *Ranitomeya* with blue-green dorsal stripes and quite peculiar behavior. In the present study, we describe it as a new species using morphological, morphometric, advertisement call, natural history, and genetic data from four mitochondrial loci.

## ﻿Materials and methods

### ﻿Sampling and specimen collection

Adult specimens

Thirteen adult individuals of the new species were manually collected in the RAPELD sampling module of the Comunidade de Santo Antônio (6°47'04.9"S, 69°52'00.3"W), Eiru River, tributary of the Juruá River, municipality of Eirunepé, Amazonas state, Brazil. The specimens were anesthetized and killed with 5% topical lidocaine. Muscle and liver tissue were preserved in 100% ethanol for posterior genetic analysis, whereas the specimens were fixed in 10% formalin and preserved in 70% ethanol. Specimens were sexed by the presence of vocal slits (exclusive to males) and internally by the condition of the gonads. Vouchers were deposited in the herpetological collection of the Instituto Nacional de Pesquisas da Amazônia (**INPA-H**; Manaus, Brazil) and Museu Paraense Emílio Goeldi (**MPEG**; Belém, Brazil).

#### ﻿Tadpole specimens

The tadpoles were collected at the same site as the adult individuals. They were euthanized as described above, and fixed and preserved in 5% neutral-buffered formalin. Tadpoles were deposited at **INPA-H**.

#### ﻿Ethical considerations

Protocols of collection and animal care followed the Brazilian Federal Council for Biology (resolution number 148/2012) and study was approved by the Ethics Committee on the Use of Animals of the Instituto Nacional de Pesquisas da Amazônia - CEUA-INPA (Process No. 35/2020, SEI 01280.001134/2020-63). Specimens were collected under collection permit number 13777-1, issued by the Centro Nacional de Pesquisa e Conservação de Répteis e Anfíbios of the Instituto Chico Mendes de Conservação da Biodiversidade – ICMBio.

### ﻿Morphological analyses

#### ﻿Adult morphometrics

Morphometric measurements were taken from eight adult males and five adult females of the new species, following Brown et al. (2011) [snout to vent length (**SVL**), head width (**HW**), head length (**HL**), interorbital distance (**IOD**), upper eyelid width (**UEW**), tympanum diameter (**TD**), eye-tympanum distance (**DET**), eye diameter (**ED**), body width (**BW**), knee-knee distance (**KK**), femur length (**FL**), tibia length (**TL**), foot length / Toe IV length (**FoL**), hand length / Finger III length (**HaL**), fingers I (**L1F**) and II (**L2F**) length, Finger III disc width (**W3FD**), finger width just below III (**W3F**)], Watters et al. (2016) [snout length (**SL**), eye-nostril distance (**END**), internarial distance (**IND**), tarsus length (**TaL**), arm length (**AL**), forearm length (**FAL**), Finger IV length (**L4F**); toes I (**L1T**), III (**L3T**) and V (**L5T**) length, Toe IV disc width (**W4TD**), fingers II (**W2FD**) and IV (**W4FD**) discs width, Finger IV width just below disc (**W4F**)], and Serrano-Rojas et al. (2017) [snout-nostril distance (**TSCN**), mouth-tympanic distance (**MTD**), Toe III disc width (**W3TD**), toes III (**W3T**) and IV (**W4T**) width just below disc]. Besides these, we also include Toe II length (**L2T**), toes I (**W1TD**), II (**W2TD**) and V (**W5TD**) disc width, toe I (**W1T**), II (**W2T**) and V (**W5T**) width just below disc, Finger I disc width (**W1FD**), and fingers I (**W1F**) and II (**W2F**) width just below disc. Measurements were taken to the nearest 0.01 mm using a stereomicroscope (S8APO, Leica) coupled to a camera (Leica, DFC295), except for SVL, which was measured with a digital caliper to the nearest 0.1 mm. Raw data are provided in Suppl. material 1.

#### ﻿Morphological and coloration description

The format of the description and terminology of the morphological characters follow Kok and Kalamandeen (2008) and Brown et al. (2011). Color in life was described based on photographs taken in the field, following the color catalog provided by Köhler (2012).

#### ﻿Tapdole morphology

Description of the external morphology of the *Ranitomeya* sp. nov. tadpole was based on three individuals, at stage 26, 29, and 39 (Gosner 1960). Morphometric measurements followed McDiarmid and Altig (1999) and Randrianiaina et al. (2011): total length (**TL**), body length (**BL**), tail length (**TAL**), maximum body height (**BH**), maximum body width (**BW**), body height at the nostril (**BHN**), body height at the eyes (**BHE**), body width at the nostril (**BWN**), body width at the eyes (**BWE**), tail muscle width at base (**TMW**), maximum tail height (**MTH**), dorsal fin height (**DF**), ventral fin height (**VF**), tail muscle height (**TMH**), interorbital distance (**IOD**), internarial distance (**IND**), rostro-eye distance (**RED**), rostro-nostril distance (**RND**), rostro-spiracle distance (**RSD**), eye diameter (**ED**), eye-nostril distance (**END**), spiracle length (**SL**), spiracle width (**SW**), spiracle height (**SW**), vent tube length (**VL**), oral disc width (**ODW**), anterior (upper) labium (**AL**), posterior (lower) labium (**PL**), first anterior tooth row (**A-1**), second anterior tooth row (**A-2**), medial gap in second anterior tooth row (**A-2 GAP**), first posterior tooth row (**P-1**), second posterior tooth row (**P-2**), third posterior tooth row (**P-3**), medial gap in the first posterior tooth row (**P-1 GAP**), lateral process of upper jaw sheath (**LP**), lower jaw sheath (**LJ**) and, finally, upper jaw sheath (**UJ**).

### ﻿Bioacoustics

#### ﻿Recording protocol

The advertisement calls of sixteen males of the new species were recorded using a digital recorder (PCM-D50, Sony) and unidirectional microphone (K6/ME66, Sennheiser, Germany). Air temperatures (24.3–26.1 °C) and humidity (89–98%) during call recording were measured with a thermohygrometer (7663.02.0.00, Incoterm). Each calling male was recorded for two min using frequency rate of 16 kHz and 16 bits of resolution in mono.

#### ﻿Data deposition

The recordings were deposited in the Fonoteca Neotropical Jacques Vielliard of the Universidade de Campinas (**FNJV**; Campinas, Brazil) under access number FNJV 124331 to 124339.

#### ﻿Analyses

Bioacoustic variables were analyzed using Raven Pro 1.6 (Bioacoustics Research Program 2014) with the following configuration: window = Blackman, Discrete Fourier Transform = 2,048 samples and 3 dB filter bandwidth = 80.0 Hz. The following temporal and spectral traits were measured: call duration (**CD**), number of notes per call (**NN**), silence between calls (**SBC**), note duration (**ND**), silence between notes (**SBN**), and minimum (**LF**), maximum (**HF**) and dominant frequency (**DF**). Dominant frequency was measured using the *Peak frequency* function; maximum and minimum frequencies were measured at 20 dB below the peak frequency to avoid background noise interference. Call description follows the call-centered approach of Köhler et al. (2017). The spectrogram and oscillogram were generated in R environment (R Core Team 2019) via the *seewave* package 2.0.5 (Sueur et al. 2008) using a Blackman window, 256 points of resolution (Fast Fourier Transform) and an overlap of 85%. The raw bioacoustic data are provided in Suppl. material 2.

### ﻿Molecular and phylogenetic analyses

#### ﻿DNA extraction and amplification

Genomic DNA was extracted from ten adult specimens (liver or muscle tissues) from both localities (Suppl. material 3). Genomic DNA was extracted using PureLink™ Genomic DNA (Invitrogen by Thermo Fisher Scientific, Carlsbad, CA, USA). Sequences of four mitochondrial loci [16S rRNA (all specimens), 12S rRNA (6 specimens), cytochrome C oxidase sub-unit 1 – CO1 (six specimens) and cytochrome *b* – cyt-*b* (4 specimens)] were amplified via polymerase chain reaction (PCR) with a general final volume of 15 μL containing 1.5 μL of 25 mM MgCl_2_, 1.5 μL of 10 mM dNTPs (2.5 mM each dNTP), 1.5 μL of buffer 10× (75 mM Tris HCl, 50 mM KCl, 20 mM (NH_4_)_2_SO_4_), 1.5 μL of forward primer (2 μM), 1.5 μL of reverse primer (2 μM), 6.4 μL of ddH_2_O and 0.1 μL of 1 U Taq DNA polymerase and 1 μL of DNA (30–50 ng/μL). For 12S, we used 12S L13 (5’- TTAGAAGAGGCAAGTCGTAACATGGTA-3’; Feller and Hedges 1998) and 12S Titus I (5’-GGTGGCTGCTTTTAGGCC-3’; Titus and Larson 1996) primers with the following PCR program: 90 s at 94 °C followed by 35 cycles at 94 °C (45 s), 55 °C (45 s) and 72 °C (90 s), and a final extension of 7 min at 72 °C. For 16S, we used 16Saf (5’-CGCCTGTTTATCAAAAACAT-3’) and 16Sbr (5’-CCGGTCTGAACTCAGATCACGT-3’) (Palumbi 1996) primers with the following PCR program: 90 s at 94 °C followed by 35 cycles at 94 °C (45 s), 55 °C (45 s) and 72 °C (90 s), and a final extension of 7 min at 72 °C. For COI, we used Chmf4f (5’-TYTCWACWAAYCAYAAAGAYATCGG-3’) and Chmr4r (5’-ACYTCRGGRTGRCCRAARAATCA-3’) (Che et al. 2012) primers with the following PCR program: 60 s at 94 °C followed by 35 cycles at 94 °C (20 s), 50 °C (50 s) and 72 °C (90 s), and a final extension of 10 min at 72 °C. Finally, for cyt-*b*, we used MVZ 15-L (5’-GAACTAATGGCCCACACWWTACGNAA-3’; Moritz et al. 1992) and H15149 (5’-AAACTGCAGCCCCTCAGAAATGATATTTGTCCTCA-3’; Kocher et al. 1989) primers with the following PCR program: 120 s at 95 °C followed by 35 cycles at 95 °C (30 s), 45 °C (60 s) and 72 °C (90 s), and a final extension of 6 min at 72 °C. All the PCR products were visualized in 1% agarose with SYBRSafe (Life Inc.) and purified using the PEG 8000 protocol (Sambrook and Russell 2001) and then submitted to sequencing using the standard protocols of the Big Dye^TM^ Terminator kit (Applied Biosystems, Inc., Grand Island, NY, USA). Forward and reverse amplicons were sequenced in a genetic analyzer (ABI PRISM 3500xL, Thermo Fisher).

#### ﻿Sequence processing

The sequences were subjected to BLAST searches (Altschul et al. 1997) in GenBank to verify whether the target had been amplified, and its quality was checked manually. The consensus sequences of each specimen were deposited in GenBank (Suppl. material 3). To infer the phylogenetic relationships of the new species, a data set containing homologous sequences was retrieved from GenBank (Suppl. material 3).

#### ﻿Sequence alignment

Sequences that represented all the diversity of *Ranitomeya* species were selected, preferably containing material assigned to the type series or from the type locality. Our complete dataset comprises 266 sequences of the four loci (33 for 12S, 120 for 16S, 17 for CO1, and 96 for cyt-*b*) that correspond to 120 terminals. Sequences of each locus were aligned using the MAFFT online server using the E-INS-i strategy for 12S and 16S gene and G-INS-i for CO1 and cyt-*b* (Katoh et al. 2019). The final matrix was composed of 120 terminals with a maximum of 2,419 bp (632 bp for 12s, 532 bp for 16S, 656 bp for COI, and 599 bp for cyt-*b*).

#### ﻿Species delimitation and genetic distances

The operational taxonomic units (OTUs) were delimited to confirm the candidate species as a single OTU. Three DNA-based species delimitation methods were used: (1) the pairwise distance-based method Assemble Species by Automatic Partitioning (ASAP, Puillandre et al. 2021) (2) the Bayesian implementation of the Poisson Tree Processes model approach (bPTP; Zhang et al. 2013); and (3) the Generalized Mixed Yule Coalescent method (single threshold GMYC; Pons et al. 2006; Monaghan et al. 2009). All methods were performed with the 16S locus, and additionally ASAP was performed with the 12S and CYTB loci to confirm the delimitation of the new species. OTUs were defined by the majority-rule consensus of the three partitions obtained with 16S locus (i.e., a lineage is considered as an OTU when it appeared in at least two out of the three results). The pairwise interspecific and the intraspecific genetic distances (p-distance and Kimura-2-parameter; Kimura 1980) using pairwise deletion were calculated between the populations of new species and close relatives using MEGA 11 (Tamura et al. 2021).

#### ﻿Phylogenetic tree reconstruction

The phylogenetic analyses were performed with Bayesian inference (BI) using the complete matrix for the four loci via the software Beast 2.6.6 (Bouckaert et al. 2014). Coding loci were partitioned to independently analyze each codon position. Two independent runs on 5×10^7^ generations of the MCMC were conducted, with sampling every 5,000 generations. The best nucleotide substitution model was selected using *bModelTest* using the “named extended models” parameters in the MCMC (Bouckaert and Drummond 2017). The clock was set to strict clock model to estimate the evolutionary rates, and the tree prior was Yule, with other priors in default.

### ﻿Data availability and supplementary materials

Raw data are provided in Suppl. materials: morphometrics (Suppl. material 1), bioacoustics (Suppl. material 2), and gene sequences (Suppl. material 3).

## ﻿Results

### ﻿Phylogenetic relationships and genetic distances

**Figure 1.**
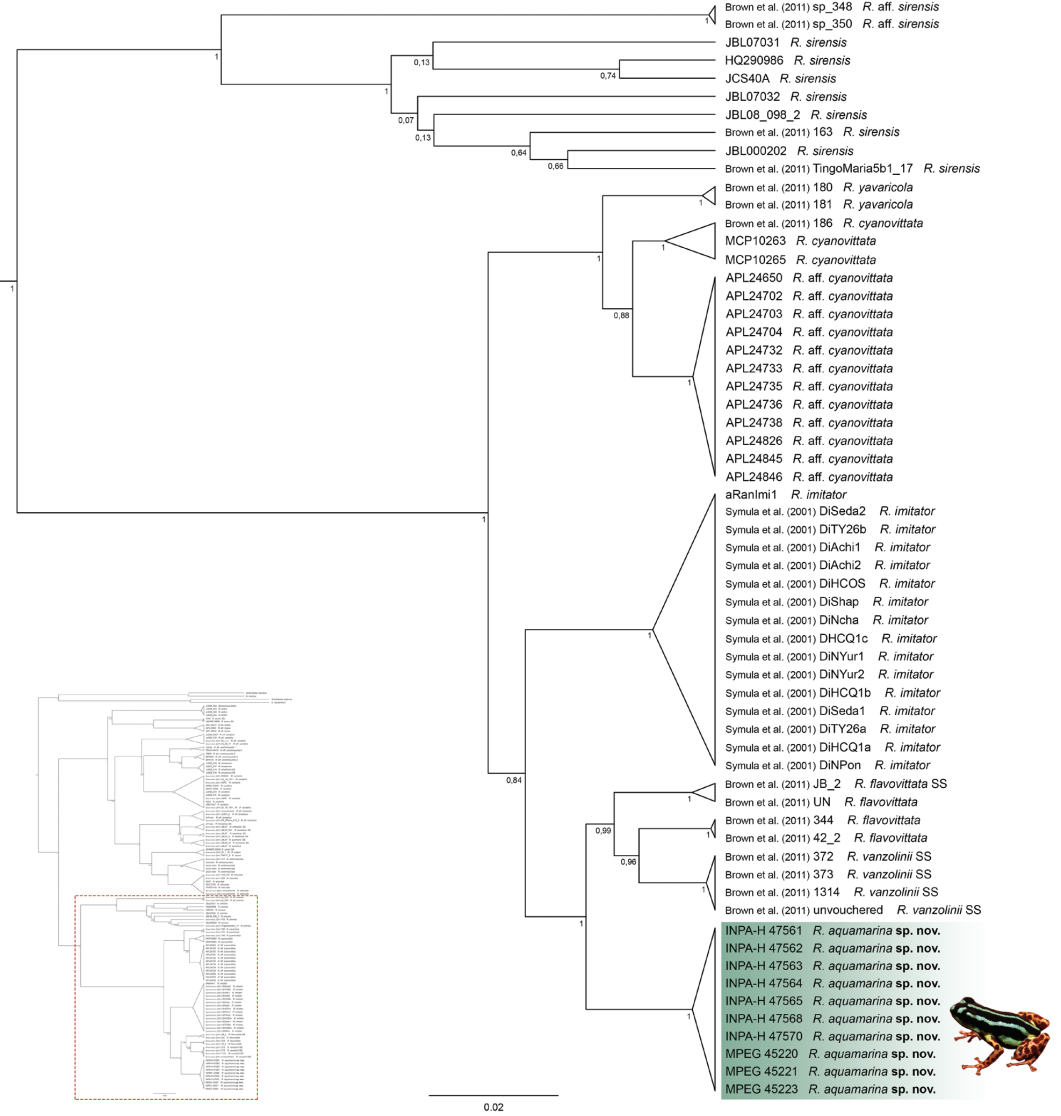
Part of the phylogenetic reconstruction showing the relationships of *Ranitomeyaaquamarina* sp. nov. Bayesian inference tree for genes 16S, 12S, COI, and cyt-*b.* Posterior probability support is shown on the branches. The species name is preceded by the specimen voucher number (continuation of the tree in Suppl. material 5).

Individuals of the new species show no intraspecific genetic variation (16S p-distance = mean 0.0%). The new species is nested within a strongly supported clade grouping *R.vanzolinii* and *R.flavovittata* (Fig. 1). This clade is sister to the clade nesting *R.imitator*. Within this large clade, interspecific p-distances range from 2.94 to 3.91% (Table 1). All these species occur in the Western Amazonian and in the Andean foothills. The new species was confirmed in all delimitation methods, including using 12S and CYTB loci (i.e., ASAP, bPTP and GMYC; Suppl. material 4).

**Table 1.** Interspecific and intraspecific genetic distances between *Ranitomeyaaquamarina* sp. nov. and closely related taxa. Uncorrected p-distances (%; lower diagonal) and Kimura-2-parameter (%; upper diagonal) for sequences in a matrix with 532 characters from 16S mtDNA gene and expressed as percentages. Numbers in bold represent intraspecific p-distance values.

Species	1	2	3	4	5	6	7	8	9	10
1. *R.aquamarina* sp. nov.	**0.00**	2.08	4.03	4.05	3.16	3.02	9.97	10.01	3.45	4.74
2. *R.cyanovittata*	3.89	**0.97**	3.13	3.96	3.76	3.86	9.14	8.99	4.06	3.63
3. R.aff.cyanovittata	3.05	2.04	**0.13**	3.13	2.26	3.16	8.16	8.00	2.55	3.27
4. R.aff.flavovittata	3.91	3.06	3.91	**0.00**	1.62	4.37	10.42	10.57	2.05	5.53
5. *R.flavovittata*	3.07	2.21	3.07	1.60	**0.25**	3.73	9.48	9.64	1.68	4.90
6. *R.imitator*	2.94	3.07	2.94	4.21	3.60	**0.60**	8.85	9.29	4.01	3.97
7. *R.sirensis*	9.19	7.67	9.19	9.61	8.78	8.26	**1.65**	5.65	9.25	6.93
8. R.aff.sirensis	9.27	7.56	9.27	9.78	8.97	8.68	5.40	**0.00**	9.91	8.01
9. *R.vanzolinii*	3.35	2.50	3.35	2.02	1.66	3.87	8.61	9.21	**0.12**	4.65
10. *R.yavaricola*	4.53	3.19	4.53	5.28	4.69	3.84	6.58	7.56	4.47	**0.24**

### ﻿Taxonomic account

#### ﻿Order Anura Fischer von Waldhein, 1813

**Family Dendrobatidae Cope, 1865 (1850)**

**Subfamily Dendrobatinae Cope, 1865 (1850)**

**Genus *Ranitomeya* Bauer, 1986**

##### ﻿ Ranitomeya aquamarina sp. nov.

Taxon classificationAnimaliaAnuraDendrobatidae

A7207489-42D4-5A0F-8C0F-2BBCEE058F6D

https://zoobank.org/16207CA5-3CDC-43FB-91E5-51139FBFD1F4

**Figure 2.**
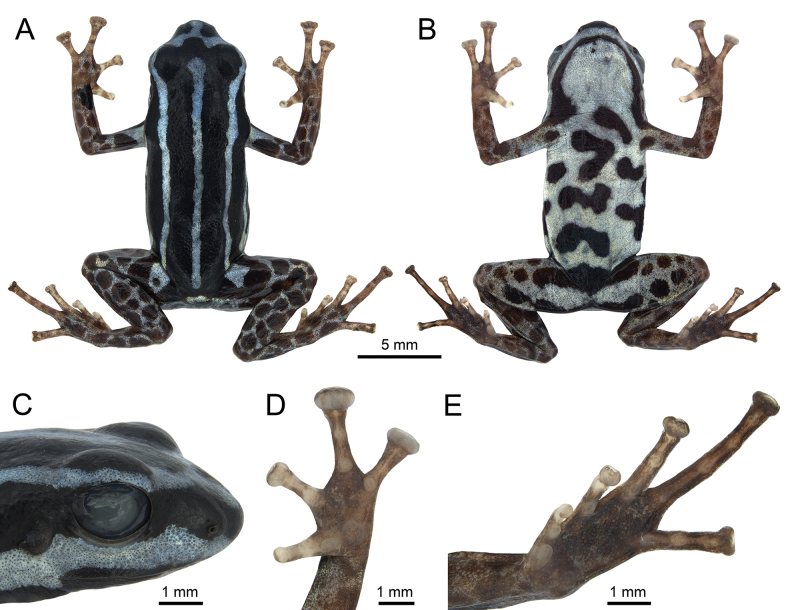
Preserved holotype of *Ranitomeyaaquamarina* sp. nov. (INPA-H 47568, field number APL 24805) from Eiru River, municipality of Eirunepé, Amazonas state, Brazil. **A** Dorsal view **B** ventral view **C** lateral head **D** hand **E** foot. Photographs ATM.

**Figure 3.**
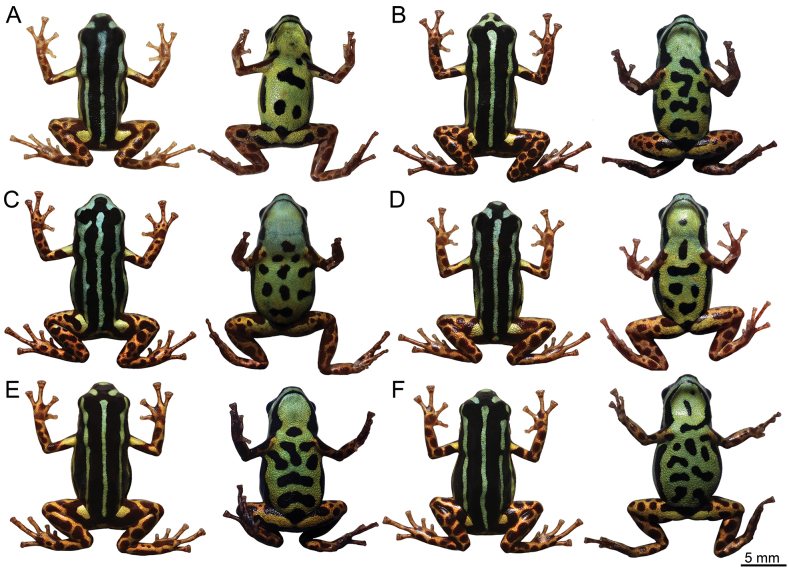
Dorsal and ventral pattern variation of *Ranitomeyaaquamarina* sp. nov. in life. Males: **A**INPA-H 47566 **B**INPA-H 47568 [Holotype] **C**MPEG 45223 **D**INPA-H 47570; and Females: **E**INPA-H 47569 **F**MPEG 45222. Photographs ATM.

**Figure 4.**
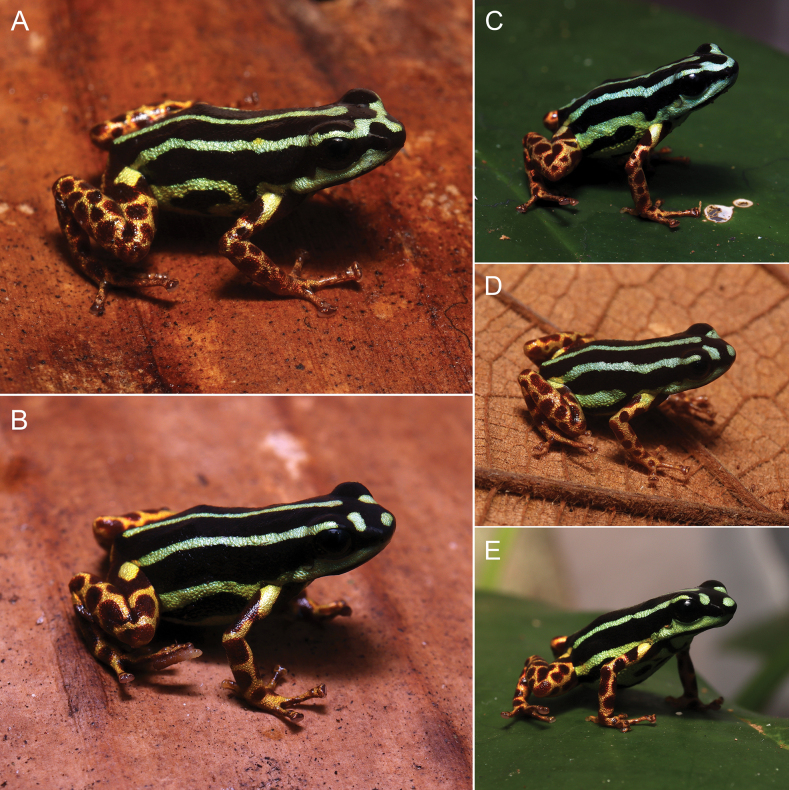
Adult individuals of *Ranitomeyaaquamarina* sp. nov. in natural posture **A** holotype, male INPA-H 47568 **B** paratype, female INPA-H 47569 **C** paratype, male MPEG 45223 **D** paratype, male INPA-H 47570 **E** Paratype, female MPEG 45222. Photographs ATM.

Figs 2 , 3 , 4 , 6 , 7 , 8 , 10 , Tables 2 , 3 , 4

###### Chresonymy.

*Ranitomeya* sp. Envira – Twomey et al. (2023); Ranitomeyaaff.sirensis – Lima et al. (2024).

###### Vernacular names.

Suggested English name: Metallic poison frog.

Suggested Spanish name: Rana venenosa metálica.

Suggested Portuguese name: Rãzinha-venenosa-metalizada.

###### Type material.

***Holotype.*** • INPA-H 47568 (field number APL 24805; Fig. 2), adult male collected by Alexander Tamanini Mônico and Albertina Pimentel Lima on 15 March 2024, from RAPELD sampling module of the Eiru River, tributary of the Juruá River, municipality of Eirunepé, Amazonas state, Brazil (6°47'04.9"S, 69°52'00.3"W, WGS84, 137 m elevation). ***Paratypes.*** Twelve adult specimens (7 males and 5 females), same locality as holotype • one male [INPA-H 47561; field number APL 24481] collected on 26 February 2023 by A.P. Lima • 3 males [INPA-H 47563, INPA-H 47564 and MPEG 45220; field numbers APL 24765, 24766 and 24768, respectively] and 3 females [INPA-H 47562, INPA-H 47565 and MPEG 45221; field numbers APL 24764, 247667 and 24769, respectively] collected on 24 March 2023 by A.P. Lima and J. Dayrell • 3 males [INPA-H 47566, MPEG 45223 and INPA-H 47570; field numbers APL 24800, 24808 and 24809, respectively] and 2 females [INPA-H 47569 and MPEG 45222; field numbers APL 24806 and 24807, respectively) collected on 14–15 March 2024 by A.T. Mônico and A.P. Lima.

###### Generic placement.

We assign the new species to *Ranitomeya*, based on the phylogenetic placement (Fig. 1) and the following external characteristics: coloration is bright and aposematic, finger I is greatly reduced and shorter than finger II, finger discs two and four are greatly expanded, dorsal skin texture is smooth (to shagreen), and toe webbing is absent (see Brown et al. 2011; Kahn et al. 2016).

###### Characterization.

This new species of *Ranitomeya* is characterized by the following combination of characters: (1) dorsal color jet black with three parallel stripes metallic light yellowish green to metallic light turquoise-green, mid-dorsal stripe extending from between eyes to slightly before the vent, dorsolateral stripes extending from the snout to the groin, where they become medium sulfur yellow; (2) venter jet black with metallic olive-yellow to metallic light yellowish green reticulations on belly, and gular region metallic light yellowish green to olive-yellow; ventrolateral stripes light yellowish green; extending from through the loreal region, to the thighs integrating into the ventral reticulate pattern, becoming medium sulfur yellow on the arms; (3) limbs medium metallic chrome orange with dark carmine spotting, presence of a conspicuous sulfur yellow spot on the dorsal surface of the thighs, forming an ‘ocellus’ like pattern; (4) dorsal skin shagreen to granular, and smooth on head; (5) gular and ventral skin shagreen to granular; (6) limbs smooth to shagreen; (7) SVL in adult males of 15.4–17.7 mm (*n* = 8) and in females of 17.3–18.5 mm (*n* = 5); (8) sexual dimorphism, females with greater SVL, BW and KK; presence of vocal slits in males, located near jaw articulation; (9) head width 0.8–1.0× body width; (10) head width 1.1–1.2× larger than head length; (11) head width 31–34% of SVL; (12) snout moderately long (SL 36–42% of HL), rounded in dorsal view and rounded to protruding in lateral view; (13) *canthusrostralis* rounded, loreal region flat; (14) nostril directed frontolaterally at the angle of the snout, internarial distance 33–39% of head width; (15) tympanum visible, tympanic membrane poorly differentiated, tympanum diameter 38–48% of eye diameter; (16) tongue ovoid, attached anteriorly; (17) dentigerous processes of vomers absent; (18) choanae ovoid and small, located marginally in the maxilla; (19) hand 24–28% of SVL, arm 25–30% of SVL; (20) fingers III > IV > II > I, Finger I 58–68% of Finger II, finger discs rounded on Finger I, and expanded and truncate on fingers III and IV; (21) thenar tubercle elliptical, palmar tubercle large and ovoid; (22) proximal subarticular tubercles ovoid, present in each finger; distal subarticular tubercle present only on Finger III; (23) knee-knee distance 80–84% of SVL, femur 94–98% of tibia; (24) toes IV > III > V > II > I, Toe I 48–64% of Toe II, finger discs not expanded and rounded on Finger I to elliptical on toes III and IV and truncate on Finger V; (25) outer metatarsal tubercle ovoid, poorly visible; inner metatarsal tubercle elliptical; (26) proximal subarticular tubercles ovoid on all toes, distal subarticular tubercles on toes III–V; (27) advertisement call with 21–45 notes and average call duration of 647–1,424 ms, note rate (28–36 notes/s) and dominant frequency of 4,996–6,288 Hz; and (28) tadpole head translucent in life, and posterior tooth rows P-1 > P-2 > P-3.

###### Differential diagnosis.

***External morphology.*** The new species differs from all currently recognized *Ranitomeya* species (*R.amazonica*, *R.benedicta*, *R.cyanovittata*, *R.defleri*, *R.fantastica*, *R.flavovittata*, *R.imitator*, *R.reticulata*, *R.sirensis*, *R.summersi*, *R.toraro*, *R.uakarii*, *R.vanzolinii*, *R.variabilis*, *R.ventrimaculata*, and *R.yavaricola*) by its unique coloration (light yellowish green to light metallic turquoise-green dorsal stripe pattern, medium metallic chrome orange limbs with dark carmine spotting, and conspicuous sulfur yellow ocellus-like spot on the dorsal surface of the thighs). *Ranitomeyaaquamarina* sp. nov. is generally most similar to *R.cyanovittata* and *R.yavaricola* but it can be easily distinguished from *R.cyanovittata* by light yellowish green to light metallic turquoise green dorsal stripes and medium metallic chrome orange limbs with dark carmine spotting (dorsal stripes turquoise blue, limbs with bluish reticulation and black spots; Pérez-Peña et al. 2010) and from *R.yavaricola* by light yellowish green to light metallic turquoise green dorsal stripes with no or only minor breaks and by dark carmine spotting on the limbs (dorsal stripes sage color, formed by points, that can become dashes, limbs solid bronze; Pérez-Peña et al. 2010).

In addition, *Ranitomeyaaquamarina* sp. nov. is distinguished by its smaller male SVL (15.4–17.7 mm) from *R.fantastica* (~ 20 mm; Boulenger 1884), *R.imitator* (~ 19 mm; Schulte 1986) and *R.summersi* (17.5–19.5 mm; Brown et al. 2008); and larger than *R.cyanovittata* (13.8 mm; Pérez-Peña et al. 2010), *R.sirensis* (14.7–15.4 mm; Aichinger 1991), *R.toraro* (14.8–15.6 mm; Brown et al. 2011) and *R.uakari* (14.8–15.5 mm; Brown et al. 2006); by its larger female SVL (17.3–18.5 mm) from *R.sirensis* (16.8 mm; Aichinger 1991), *R.toraro* (16.2–16.7 mm; Brown et al. 2011), *R.uakari* (15.7–16.2 mm; Brown et al. 2006); *R.yavaricola* (16.7–16.8 mm; Pérez-Peña et al. 2010); by its greater female head width (5.7–5.9 mm) from *R.cyanovittata* (5.6 mm; Pérez-Peña et al. 2010), *R.sirensis* (5.4 mm; Aichinger 1991), *R.toraro* (5.0–5.3 mm; Brown et al. 2011), *R.uakarii* (5.0–5.2 mm; Brown et al. 2006), *R.yavaricola* (5.1–5.7 mm; Pérez-Peña et al. 2010); by its greater male head length (4.8–5.4 mm) from *R.cyanovittata* (3.6 mm; Pérez-Peña et al. 2010) and *R.sirensis* (3.0–3.8 mm; Aichinger 1991), but smaller than *R.imitator* (~ 6 mm) and *R.yavaricola* (5.5–6.6 mm; Pérez-Peña et al. 2010); by its smaller female head length (5.1–5.2 mm) from *R.toraro* (5.5 mm; Brown et al. 2011) and *R.yavaricola* (5.9–6.3 mm; Pérez-Peña et al. 2010) and larger than *R.sirensis* (4.0 mm; Aichinger 1991).

***Bioacoustics.*** The advertisement call of *R.aquamarina* sp. nov. is distinguished by its longer call duration (647–1,424 ms) from the call of *R.amazonica* (160–360 ms; Brown et al. 2011), *R.benedicta* (100–170 ms; Brown et al. 2008), *R.defleri* (410–620 ms; Twomey and Brown 2009), *R.fantastica* (180–320 ms; Brown et al. 2011), *R.reticulata* (180–290 ms; Brown et al. 2011), *R.summersi* (380–500 ms; Brown et al. 2008), *R.uakarii* (260–290 ms; Brown et al. 2006, Brown et al. 2011), *R.vanzolinii* (570–640 ms; Brown et al. 2011), *R.variabilis* (140–440 ms; Brown et al. 2011) and *R.ventrimaculata* (320–380 ms; Brown et al. 2011). Furthermore, it differs by its higher dominant frequency (4,996–6,288 Hz) from the calls of *R.benedicta* (3,190–4,240 Hz; Brown et al. 2008), *R.fantastica* (2,950–3,790 Hz; Brown et al. 2011), *R.reticulata* (4,140–4,480 Hz; Brown et al. 2011), *R.summersi* (2760–3220 Hz; Brown et al. 2008), *R.uakarii* (3,790–4,130 Hz; Brown et al. 2006, Brown et al. 2011), and *R.ventrimaculata* (4190–4400 Hz; Brown et al. 2011); by its lower number of notes (21–45) from the calls of *R.reticulata* (48–94; Brown et al. 2011) and *R.ventrimaculata* (58–63; Brown et al. 2011); by greater number of notes from the calls of *R.fantastica* (10–13; Brown et al. 2011), *R.summersi* (14–16; Brown et al. 2008), *R.uakarii* (14–16; Brown et al. 2006, Brown et al. 2011), and *R.vanzolinii* (16–17; Brown et al. 2011); by its smaller note rate (28–36 notes/s) from the calls of *R.amazonica* (85–138 notes/s; Brown et al. 2011), *R.defleri* (94–104 notes/s; Twomey and Brown 2009), *R.fantastica* (41–57 notes/s; Brown et al. 2011), *R.reticulata* (270–382 notes/s; Brown et al. 2011), *R.summersi* (39–40 notes/s; Brown et al. 2008), *R.uakarii* (50–58 notes/s; Brown et al. 2006; Brown et al. 2011), *R.variabilis* (106–297 notes/s; Brown et al. 2011), and *R.ventrimaculata* (166–181 notes/s; Brown et al. 2011).

The advertisement call of *R.aquamarina* sp. nov. is highly similar to the calls of all other species of the *R.vanzolinii* group, which have long-lasting trills, but it can still be distinguished from the call of *R.vanzolinii*, which has slightly lower note rate of 26–28 notes/s (Brown et al. 2011). On the other hand, based on the literature, the call of *R.aquamarina* sp. nov. is indistinguishable from the calls of *R.flavovittata*, *R.imitator*, *R.sirensis*, and *R.yavaricola* (Perez-Peña et al. 2010; Brown et al. 2011). There is no available information about minimum and maximum frequencies, note duration and inter-notes interval in these species. In addition, the calls of *R.toraro* and *R.cyanovittata* remain completely unknown.

***Tadpole morphology.*** There is little information available about the tadpoles of the *Ranitomeya* species, but we did find information for ten species (*R.amazonica*, *R.benedicta*, *R.defleri*, *R.flavovittata*, *R.imitator*, *R.reticulata*, *R.toraro*, *R.uakarii*, *R.vanzolinii* and *R.variabilis*). The tadpoles of *R.aquamarina* sp. nov. differ from the tadpoles of all these species by absence of emarginate marginal papillae.

Because there is great variation between the initial (25–27), intermediate (28–32), and final (37–40) stages, we compared them using the ratios between measurements (the characteristics of compared species are given in parentheses). The labial tooth row formula in *R.aquamarina* sp. nov. is 2(2)/3(1) in all stages and differs from the formula of *R.toraro* 2(2)/2(1) (Brown et al. 2011). The ratios (in percentages) tail length/total length are in *R.aquamarina* sp. nov. (63 to 64% in all stages) greater than in *R.amazonica* (45% st. 29, Brown et al. 2011), *R.flavovittata* (57% st. 26; Brown et al. 2011), *R.imitator* (62% st. 26; Brown et al. 2011), *R.reticulata* (41% st. 30; Brown et al. 2011), *R.uakarii* (62%, st. 29; Brown et al. 2011) and *R.variabilis* (37% st. 28; Brown et al. 2011), and smaller than in *R.toraro* (64.2% st. 25; Brown et al. 2011), *R.vanzolinii* (67.9% st. 38; Brown et al. 2011) and *R.yavaricola* (64.4% st. 25; Pérez-Peña et al. 2010).The ratios oral disc width/body width are in *R.aquamarina* sp. nov. (42% at stage 26, 36% at stage 29, and 35% at stage 39) greater than in *R.amazonica* (29% st. 26, 22% st. 29, 33% st. 38; Brown et al. 2011, Klein et al. 2020), *R.flavovittata* (28% st. 26; Brown et al. 2011), *R.imitator* (38% st. 26; Brown et al. 2011), *R.toraro* (36% st. 25; Brown et al. 2011), *R.reticulata* (14% st. 30; Brown et al. 2011), *R.uakarii* (35%, st. 29; Brown et al. 2011), and smaller than in *R.vanzolinii* (38.9% st. 38; Brown et al. 2011).

The ratios tail muscle width/tail muscle height are in *R.aquamarina* sp. nov. (91% at stage 26 and 115% at stage 29) greater than in *R.flavovittata* (63% st. 26; Brown et al. 2011), *R.imitator* (52% st. 26; Brown et al. 2011), *R.amazonica* (76% st. 29; Brown et al. 2011), *R.reticulata* (92% st. 30; Brown et al. 2011), *R.uakari* (88%, st. 29; Brown et al. 2011), and *R.variabilis* (72% st. 30; Brown et al. 2011), and smaller than in *R.toraro* (100% st. 25; Brown et al. 2011).

Posterior tooth row formula of *R.aquamarina* sp. nov. (P-1 > P-2 > P-3 in all stages) differs from the formulas of all other described tadpoles: *R.amazonica* (P-1 = P-2 > P-3, P-3 = 80% of P-1; Brown et al. 2011), *R.benedicta* (P-1 = P-2 = P-3; Klein et al. 2020), *R.flavovittata* (P-1 = P-2 > P-3, P-3 = 80% of P-1; Brown et al. 2011), *R.imitator* (P-1 = P-2 > P-3, P-3 = 55% of P-1; Brown et al. 2011; Klein et al. 2020), *R.reticulata* (P-1 = P-2 > P-3, P-3 = 80% of P-1; Brown et al. 2011; Klein et al. 2020), *R.toraro* (P-1 > P-2; Brown et al. 2011), *R.uakarii* (P-1 = P-2 > P-3, P-3 = 75% of P-1 and P-2; Brown et al. 2011), *R.vanzolinii* (P-1 < P-2 = P-3, P-1 = 44.6% of P-2; Brown et al. 2011), *R.variabilis* (P-1 = P-2 > P-3, P-3 = 75% of P-1; Brown et al. 2011) and *R.yavaricola* (P-1 = P-2 > P-3; Pérez-Peña et al. 2010).

In life, tadpoles of *R.aquamarina* sp. nov. have a translucent brownish head in all stages, which differs from all the other tadpoles described: *R.amazonica* (head and body black to gray; Brown et al. 2011, Klein et al. 2020), *R.benedicta* (head and body dark gray, with a reddish area anterior and posterior to the eye; Klein et al. 2020), *R.imitator* (head beige strongly dotted with yellowish green spots; Klein et al. 2020), *R.reticulata* (head and body gray; Brown et al. 2011), and *R.toraro* (head and body gray; Brown et al. 2011), *R.uakari* (head gray; Brown et al. 2011), *R.vanzolinii* (head and body dark gray to black; Klein et al. 2020), *R.variabilis* (head and body gray; Brown et al. 2011) and *R.yavaricola* (light grey; Pérez-Peña et al. 2010). When preserved, the tadpoles of *R.aquamarina* sp. nov. are cream with brown reticules on the lateral, dorsal, anterior half belly, spiracles, tail muscle and fins and differ from *R.amazonica* (dorsum dark gray and hindlimbs bluish gray, spotted with dark dots; Klein et al. 2020), *R.benedicta* (dorsum and hindlimbs dark gray; Klein et al. 2020), *R.imitator* (beige, densely spotted with gray dots; Klein et al. 2020) and *R.vanzolinii* (dorsum of body grayish brown, tail musculature light yellowish brown and fins translucent; Brown et al. 2011).

###### Holotype description.

Adult male (INPA-H 47568, field number APL 24805, Figs 2–4). SVL 17.1 mm; head width slightly smaller than body width; head width larger than head length; head width 30% of SVL (Fig. 2A, B). Snout rounded in dorsal view and rounded to protruding in lateral view (Fig. 2C). Nostril directed frontolaterally at the angle of the snout, 1.0 mm from the tip of the snout; internarial distance 2.0 mm, 35.6% of head width. *Canthusrostralis* rounded, loreal region flat. Eye-nostril distance 1.5 mm, 74.0% of horizontal eye diameter. Tympanic annulus and tympanic membrane present. Tympanum slightly ovoid, posterodorsal margin hidden by depressor muscle, tympanum 44.4% of eye diameter. Tongue ovoid, attached anteriorly, longer than wide, median lingual process absent. Dentigerous processes of vomers absent. Choanae ovoid and small (0.4 mm), located marginally in the maxilla, not visible in ventral view. Paired vocal slits present, located near jaw articulation.

Forelimbs slender, hands relatively large, 25.9% of SVL. Finger I shorter (66.9%) than Finger II; Finger III > IV > II > I. Discs on fingers III and IV considerably expanded and truncate, disc of Finger II moderately expanded and elliptical, disc of Finger I rounded. Ulnar tubercles absent. Hands lacking lateral fringes and webbing. Palmar tubercle rounded, unpigmented, ~ 4× larger than the subarticulars. Thenar tubercle elliptical, small. Large unpigmented, rounded, proximal subarticular tubercles present on base of each finger. Rounded distal subarticular tubercle visible only on Finger III (Fig. 2D).

Length of legs moderate, femur slightly smaller than tibia, with 93.7% of the tibia length; knee-knee distance 80% of SVL. Relative lengths of appressed toes IV > III > V > II > I. First toe short, Toe I disc not expanded and rounded, Toe II with slightly expanded and rounded disc, toes III–V with moderately expanded discs, III and IV elliptical, and V truncated. Tarsal tubercle absent; feet lacking webbing; lateral fringes poorly developed. Outer metatarsal tubercle ovoid, unpigmented, poorly visible. Inner metatarsal tubercle elliptical, unpigmented. Proximal subarticular tubercles present at base of each toe, large and ellipticals on toes I and II, small and rounded on toes III–V, all unpigmented. Distal subarticular tubercles large on toes III and V, and poorly distinguished on Toe IV. Two medial subarticular tubercles diffused on Toe IV (Fig. 2E). Holotype measurements summarized in Table 2.

**Table 2.** Morphometric measurements (mm) of adult type specimens of *Ranitomeyaaquamarina* sp. nov. Values express mean ± standard deviation, and range.

Morphometric measurements	Holotype	Males (*n* = 7)	Females (*n* = 5)
SVL – Snout to vent length	17.1	16.9 ± 0.73 (15.4–17.7)	17.9 ± 0.45 (17.3–18.5)
HL – Head length	5.1	5.1 ± 0.18 (4.8–5.4)	5.2 ± 0.05 (5.1–5.2)
HW – Head width	5.7	5.6 ± 0.34 (4.9–5.9)	5.8 ± 0.08 (5.7–5.9)
IOD – Interorbital distance	2.3	2.4 ± 0.10 (2.2–2.5)	2.3 ± 0.13 (2.2–2.5)
UEW – Upper eyelid width	1.4	1.4 ± 0.12 (1.2–1.6)	1.6 ± 0.08 (1.5–1.7)
MTD – Mouth-tympanum distance	0.8	0.7 ± 0.07 (0.5–0.7)	0.8 ± 0.08 (0.7–0.9)
TD – Tympanum diameter	0.9	0.9 ± 0.06 (0.8–1.0)	1.0 ± 0.09 (0.9–1.1)
DET – Distance from eye to tympanum	0.6	0.6 ± 0.04 (0.6–0.7)	0.7 ± 0.05 (0.6–0.7)
ED – Eye diameter	1.0	2.0 ± 0.11 (1.8–2.2)	2.1 ± 0.09 (2.0–2.2)
SL – Snout length	2.0	1.9 ± 0.10 (1.7–2.0)	2.1 ± 0.11 (1.7–2.2)
END – Eye-nostril distance	1.5	1.4 ± 0.07 (1.3–1.6)	1.5 ± 0.07 (1.4–1.6)
BW – Body width	5.8	5.6 ± 0.28 (5.1–5.9)	6.6 ± 0.36 (6.2–7.1)
TSCN – Snout-nostril distance	1.0	0.9 ± 0.07 (0.8–1.0)	1.1 ± 0.02 (1.1–1.1)
IND – Internarial distance	2.0	1.9 ± 0.17 (1.6–2.1)	2.2 ± 0.09 (2.1–2.3)
KK – Knee-knee distance	13.7	13.8 ± 0.46 (12.9–14.3)	14.7 ± 0.33 (14.2–15.0)
FL – Femur length	6.6	6.9 ± 0.21 (6.6–7.2)	7.2 ± 0.14 (7.0–7.4)
TL – Tibia length	7.0	6.9 ± 0.37 (6.3–7.3)	7.5 ± 0.18 (7.2–7.6)
TaL – Tarsus length	3.7	4.0 ± 0.32 (3.6–4.4)	4.4 ± 0.16 (4.1–4.6)
FoL – Foot length	7.1	6.7 ± 0.37 (6.0–7.1)	6.8 ± 0.38 (6.3–7.2)
LT1 – Toe I length	1.9	1.7 ± 0.17 (1.5–2.0)	1.9 ± 0.12 (1.7–2.0)
LT2 – Toe II length	3.0	3.0 ± 0.16 (2.8–3.3)	3.1 ± 0.21 (2.9–3.5)
LT3 – Toe III length	4.8	4.9 ± 0.30 (4.4–5.3)	5.1 ± 0.29 (4.6–5.3)
LT4 – Toe IV length	7.1	6.7 ± 0.37 (6.0–7.1)	6.8 ± 0.38 (6.3–7.2)
LT5 – Toe V length	4.8	4.4 ± 0.44 (3.9–4.9)	4.8 ± 0.25 (4.4–5.1)
W1TD – Width of disc on Toe I	0.3	0.4 ± 0.04 (0.3–0.4)	0.4 ± 0.06 (0.3–0.5)
W1T – Width of Toe I just below disc	0.3	0.3 ± 0.04 (0.3–0.4)	0.4 ± 0.04 (0.3–0.4)
W2TD – Width of disc on Toe II	0.6	0.5 ± 0.06 (0.4–0.6)	0.6 ± 0.04 (0.5–0.6)
W2T – Width of Toe II just below disc	0.4	0.4 ± 0.04 (0.4–0.5)	0.5 ± 0.02 (0.4–0.5)
W3TD – Width of disc on Toe III	0.7	0.7 ± 0.08 (0.5–0.8)	0.7 ± 0.09 (0.6–0.8)
W3T – Width of Toe III just below disc	0.5	0.5 ± 0.05 (0.5–0.6)	0.6 ± 0.06 (0.5–0.6)
W4TD – Width of disc on Toe IV	0.8	0.8 ± 0.09 (0.7–0.9)	0.9 ± 0.11 (0.7–1.0)
W4T – Width of Toe IV just below disc	0.6	0.7 ± 0.08 (0.5–0.7)	0.7 ± 0.08 (0.6–0.8)
W5TD – Width of disc on Toe V	0.7	0.8 ± 0.08 (0.6–0.8)	0.8 ± 0.11 (0.7–1.0)
W5T – Width of Toe V just below disc	0.6	0.7 ± 0.07 (0.5–0.7)	0.7 ± 0.08 (0.6–0.8)
AL – Arm length	4.5	4.8 ± 0.23 (4.4–5.0)	5.1 ± 0.19 (4.8–5.3)
FAL – Forearm length	4.1	4.1 ± 0.12 (3.9–4.3)	4.2 ± 0.10 (4.1–4.4)
HaL – Hand length	4.4	4.5 ± 0.27 (4.0–4.7)	4.6 ± 0.16 (4.5–4.8)
L1F – Finger I length	2.1	1.9 ± 0.08 (1.8–2.1)	2.1 ± 0.07 (2.0–2.2)
L2F – Finger II length	3.1	3.2 ± 0.21 (2.8–3.5)	3.3 ± 0.19 (3.1–3.5)
L3F – Finger III length	4.4	4.5 ± 0.27 (4.0–4.7)	4.6 ± 0.16 (4.5–4.8)
L4F – Finger IV length	3.5	3.5 ± 0.26 (3.0–3.8)	3.7 ± 0.13 (3.5–3.8)
W1FD – Width of disc on Finger I	0.4	0.4 ± 0.07 (0.4–0.6)	0.4 ± 0.03 (0.4–0.5)
W1F – Width of Finger I just below disc	0.4	0.4 ± 0.06 (0.3–0.5)	0.4 ± 0.04 (0.3–0.4)
W2FD – Width of disc on Finger II	0.7	0.7 ± 0.08 (0.6–0.8)	0.8 ± 0.14 (0.7–1.0)
W2F – Width of Finger II just below disc	0.6	0.5 ± 0.08 (0.4–0.7)	0.6 ± 0.06 (0.5–0.7)
W3FD – Width of disc on Finger III	1.1	0.9 ± 0.09 (0.8–1.1)	0.9 ± 0.10 (0.8–1.0)
W3F – Width of Finger III just below disc	0.7	0.7 ± 0.08 (0.6–0.8)	0.7 ± 0.09 (0.6–0.8)
W4FD – Width of disc on Finger IV	0.9	0.9 ± 0.07 (0.8–0.9)	0.9 ± 0.09 (0.7–1.0)
W4F – Width of Finger IV just below disc	0.7	0.7 ± 0.06 (0.6–0.7)	0.7 ± 0.04 (0.6–0.7)

Skin texture nearly smooth to shagreen on head, becoming weakly granular on the dorsum and limbs. Ventral surface of limbs smooth to shagreen. Gular region and venter shagreen. Arms smooth to shagreen.

In life, dorsal surface jet black (color 300 by Köhler 2012) with three parallel metallic pale yellowish green stripes (color 100 by Köhler 2012) (Figs 3B, 4A), middorsal stripe extends from between the eyes to slightly before the vent. Dorsolateral stripes extend from the snout, where they merge, to the groin. Slightly before the groin, the dorsolateral stripes become metallic light sulfur yellow (color 93 by Köhler 2012), and merge with a medium sulfur yellow (color 94 by Köhler 2012) spot on the dorsal surface of the thigh. Ventrolateral stripes metallic light yellowish green (color 100 by Köhler 2012), extending through the loreal region, without touching the upper labium, to the thighs and integrating into the ventral reticulate pattern; its color leaks slightly on the arms, becoming medium sulfur yellow (color 94 by Köhler 2012) and integrating into the arms reticulate pattern. On the side of the head, the stripe does not reach the nostril, eye, and tympanum. Venter jet black (color 300 by Köhler 2012) with metallic olive-yellow (color 117 by Köhler 2012) to metallic light yellowish green (color 100 by Köhler 2012) reticulations on belly. Gular region fully metallic light yellowish green (color 100 by Köhler 2012; Fig. 3B). Both forelimbs and hindlimbs medium metallic chrome orange (color 75 by Köhler 2012) with dark carmine spots (color 61 by Köhler 2012) in the ventral surface of the thighs proximal to the body. Iris jet black (color 300 by Köhler 2012).

After four months in alcohol, general color pattern remained, but colors faded (Fig. 2A, B). Stripes and limb reticulations become pale cyan (color 157 by Köhler) and ventral surfaces cyan-white (color 156 by Köhler). Forelimb and hindlimb spots become raw umber (color 280 by Köhler 2012).

###### Variation.

SVL ranges from 15.4 to 17.7 mm in males (*n* = 7) and from 17.3 to 18.5 mm in females (*n* = 5) (Table 2). The dorsal stripe pattern is very consistent among individuals (Figs 3, 4). The middorsal stripe is complete and extends from between the eyes to slightly before the vent, except for one individual, where the stripe interrupted in the scapular region. Dorso-lateral stripes are complete and extend from eyes to the groin (Fig. 4), except for two individuals, where the stripes are interrupted in the arm region (unilaterally or bilaterally).

**Figure 5.**
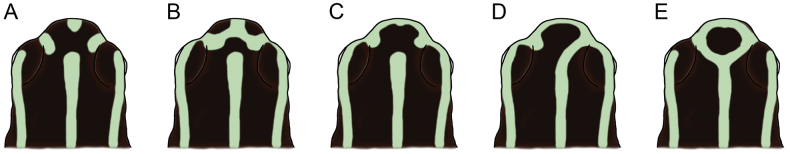
Schematic illustration of the head patterns in adult individuals of *Ranitomeyaaquamarina* sp. nov.

The head stripes have five patterns in the type series (Fig. 5). The basis is formed by three dots: one on the tip of the snout and another on the underside of each eye. These dots can be connected with the dorsal stripes. Three of the five patterns found do not have the dots connected with the middorsal stripe (Fig. 5A–C), while the other two do (Fig. 5D, E). The most common pattern presents the three unconnected dots (*n* = 53.8%; Fig. 5A), followed by the pattern of three dots connected with dorsolateral stripes forming a W-shape (Fig. 5B) and three dots connected in the margin of the snout with one of the lateral dots connected with the mid-dorsal stripe (Fig. 5D) (*n* = 15.4% each). The less frequent patterns are formed by three dots connected at the margin of the snout (Fig. 5C) or connected with the mid-dorsal stripe forming an O-shape (Fig. 5E) (*n* = 7.7% each).

The coloration of the stripes varies from metallic light yellowish green (color 100 by Köhler 2012) to metallic pale turquoise green (color 146 by Köhler 2012; Fig. 3). All individuals show a medium sulfur yellow (color 94 by Köhler 2012) spot in the dorsal surface of the thigh, well-defined in most of them (*n* = 84,6%; Fig. 3). Limb coloration is very constant, but the spots can vary in their size and quantity, going from denser, small, rounded spots to less dense large merging spots.

###### Advertisement call.

**Figure 6.**
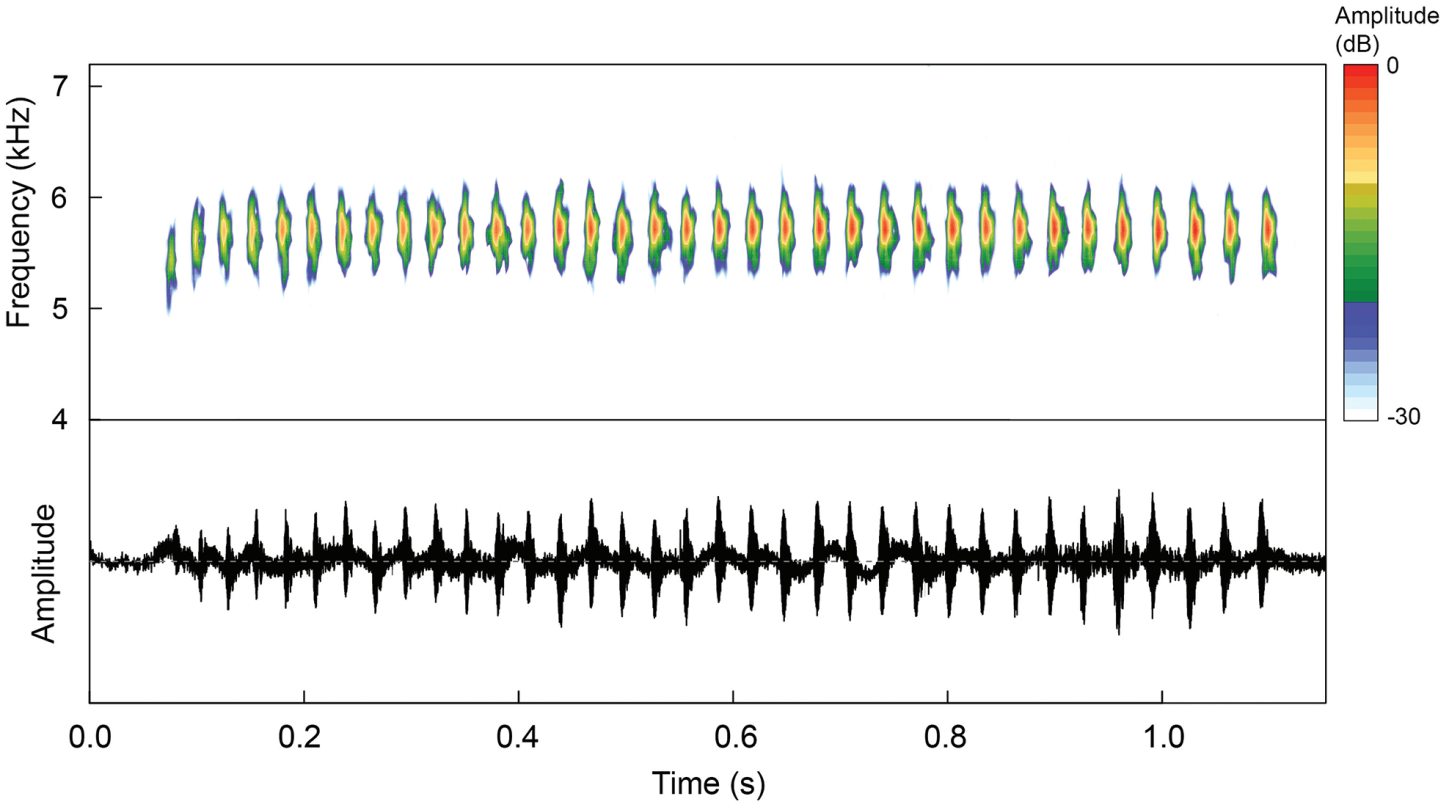
Advertisement call of the holotype (INPA-H 47568, FNJV 124337) of *Ranitomeyaaquamarina* sp. nov. recorded at the Comunidade Santo Antônio, municipality of Eurinepé, Amazonas state, Brazil. Air temperature 25.2 °C.

The advertisement call of *Ranitomeyaaquamarina* sp. nov. (*n* = 7 males) consist of a long-lasting trill of 21–45 notes (*n* = 44 calls)—most commonly of 32–38 notes (*n* = 24 calls)—a call duration of 984 ± 197 ms (647–1,424 ms)—and silence between calls of 5.8–115.3 s (most commonly between 7 and 18 s) (n = 24 silence between calls). Notes are distinct, separated by silence intervals, with note duration of 11.7 ± 0.14 ms (9.6–14.8 ms), a silence between notes of 19.4 ± 0.2 ms (15.6–22.7 ms), and a note rate of 32.8 ± 2.2 s (28–36). Calls are emitted with a minimum frequency (LF) of 5,139 ± 283 Hz (4,699–5,860 Hz), a maximum frequency (HF) of 6,054 ± 255 Hz (5,545–6,600 Hz) and a dominant frequency (DF) of 5,633 ± 289 Hz (4,996–6,288 Hz) (Fig. 6). However, the first note is emitted at approximately 300 Hz – a lower frequency compared to the subsequent notes (LF of 4,921 ± 187 Hz, HF of 5,683 ± 211 Hz and a DF of 5,340 ± 202 Hz). Temporal and spectral traits, summarized according to individual call arrangement, are presented in Table 3.

**Table 3.** Acoustic variables of the advertisement call of 44 analyzed calls of seven males of *Ranitomeyaaquamarina* sp. nov. Abbreviation: SD – standard deviation.

Variables	Mean	SD	Minimum	Maximum
CD – Call duration (ms)	984	197	647	1,424
SBC – Silence between calls (s)	19.3	20.6	5.8	115.3
NN – Number of notes per call	32.4	6.7	21	45
ND – Note duration (ms)	11.8	0.13	9.6	14.8
SBN – Silence between notes (ms)	19.4	0.19	15.6	22.7
NR – Note rate (notes per second)	32.4	2.4	28	36
LF – Minimum frequency (Hz)	5,132	272	4,699	5,860
HF – Maximum frequency (Hz)	6,059	244	5,545	6,600
DF – Dominant frequency (Hz)	5,640	277	4,996	6,288

###### Tadpole morphology.

Tadpole description is based on three specimens (vouchers INPA-H 47567) at Gosner (1960) stages 26, 29, and 39 (for measurements see Table 4). Since few tadpoles of *Ranitomeya* were described and each at a different stage, it is important to present the measurements for the three stages that were found (see Table 4).

**Table 4.** Morphometric measurements (mm) of three tadpoles of *Ranitomeyaaquamarina* sp. nov. from Eirunepé municipality, Amazonas state, Brazil.

Measurements	Tadpole stages		
	26	29	39
TL – Total length	13.7	19.9	25.0
BL – Body length	5.1	7.3	9.1
TAL – Tail length	8.6	12.7	16.0
BH – Body height	2.4	4.0	4.5
BW – Body width	3.3	5.0	6.0
BHN – Body height at the nostril	1.3	1.7	2.0
BHE – Body height at the eyes	1.8	2.6	3.4
BWN – Body width at the nostril	2.2	2.6	3.0
BWE – Body width at the eyes	3.0	4.1	4.6
TMW – Tail muscle width at base	1.1	1.9	2.1
MTH – Maximum tail height	2.3	3.6	4.5
DF – Dorsal fin height	0.6	0.9	1.3
VF – Ventral fin height	0.6	0.9	1.2
TMH – Tail muscle height	1.2	1.6	2.3
IOD – Interorbital distance	1.3	1.6	2.8
IND – Internarial distance	0.8	1.2	1.5
RED – Rostro-eye distance	1.9	2.2	2.5
RND – Rostro-nostril distance	0.9	1.0	1.0
RSD – Rostro-spiracle distance	3.5	4.4	5.7
ED – Eye diameter	0.4	0.7	0.9
END – Eye-nostril distance	0.7	0.8	0.9
SL – Spiracle length	0.5	0.7	0.9
SW – Spiracle width	0.3	0.6	0.6
SH – Spiracle height	0.7	0.7	0.9
VL – Vent tube length	0.5	1.1	-
ODW – Oral disc width	1.4	1.8	2.1
AL – Anterior (upper) labium	0.1	0.3	0.3
PL – Posterior (lower) labium	0.1	0.2	0.3
A-1 – First anterior tooth row	1.1	1.3	1.4
A-2 – Second anterior tooth row	1.2	1.4	1.5
A-2 GAP – Medial gap in second anterior tooth row	0.5	0.5	0.6
P-1 – First posterior tooth row	0.9	1.2	1.4
P-2 – Second posterior tooth row	0.9	1.1	1.2
P-3 – Third posterior tooth row	0.8	1.0	1.1
P-1 GAP – Medial gap in the first posterior tooth row	0.1	0.3	0.1
LP – Lateral process of upper jaw sheath	0.1	0.1	0.1
LJ – Lower jaw sheath	0.5	0.8	0.8
UJ – Upper jaw sheath	0.7	0.9	0.9

**Figure 7.**
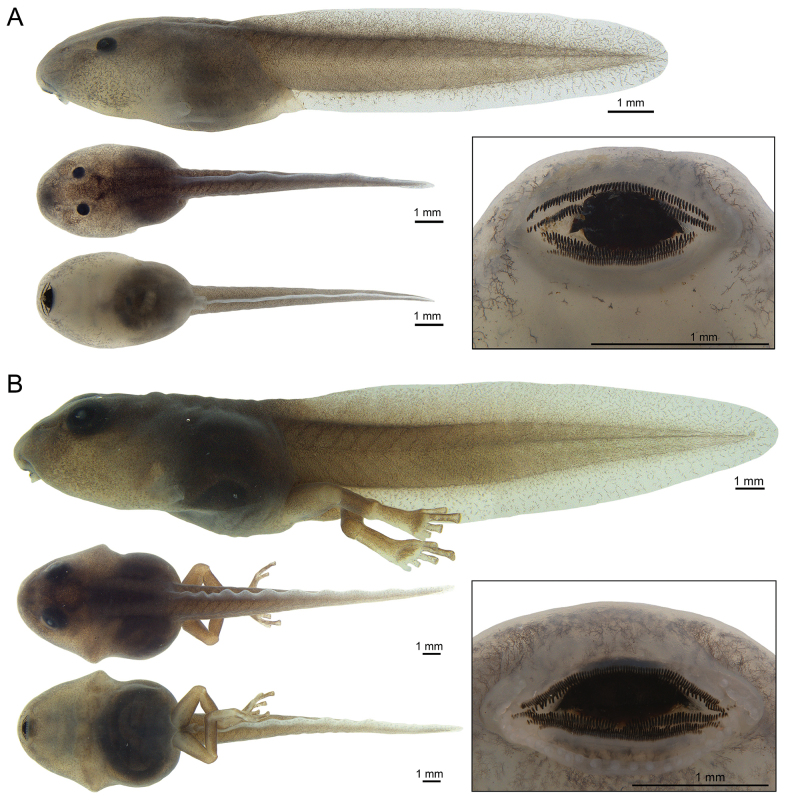
Preserved tadpoles of *Ranitomeyaaquamarina* sp. nov. (INPA-H 47567). Lateral, dorsal and ventral views of the body, and ventral view of the oral disc, respectively. **A** Gosner stage 26 **B** gosner stage 39. Photographs ATM.

Body shape in stage 26 ovoid in dorsal and lateral view (Fig. 7A). In stages 29 and 39, depressed, broadly rounded to truncate each end of body (Fig. 7B). Body length corresponds to 36.9%, 36.4%, and 36.3% of the total length, respectively. Tail length 63%, 64%, and 63% of the total length, respectively. Snout rounded in dorsal and lateral view in all stages (Fig. 7). Eyes positioned dorsally, directed dorsolaterally (Fig. 7), eye diameters stage 26 = 0.44 mm, stage 29 = 0.65 mm, and stage 39 = 0.88 mm (Table 4) correspond to 8.7%, 9.0%, and 9.6% of body length, respectively. Nostrils small and elliptical, with slightly elevated marginal rim, located dorsally in the middle between the tip of snout and eyes, in all stages, directed antero-laterally, spiracle sinistral, opening dorsoposteriorly, located well below at the middle line of the body axis, length 10.2%, 9.1% and 8.9% of body length, respectively. In all stages, the spiracle is visible dorsally, ventrally, and laterally (Fig. 7). Digestive tract dark, folded, occupies half of the belly, without visible organs. Dextral vent tube measures 0.5 mm at stage 26, 1.1 mm at stage 29, and partially absorbed at stage 39. Caudal musculature robust, tapering gradually, width at body-tail junction of 1.1 mm at stage 26, 1.9 mm at stage 29 and 2.1 mm at stage 39), tail muscle height of 1.2 mm, 1.6 mm, and 2.3 mm, respectively, not reaching the tail tip. Dorsal fin slightly higher than ventral fin, 54.7% to 57.4% of the height of the tail muscles, originating at posterior end of the body. Ventral fin 24.3% to 26.4% of tail width (Table 4). Tail tip ovoid.

Oral apparatus located antero-ventrally, not emarginated laterally. Transverse width of oral disc 42% of body width at stage 26, 36% at stage 29, and 35% at stage 39, respectively. Lower and lateral labium free from body wall. Anterior labium with groups of five or six short elliptical papillae, distributed in a single row on each side of the lateral margins and split by a medial gap. Posterior labium with a single row of marginal short elliptical papillae in all stages. Jaw sheaths oval, upper jaw sheath slightly wider than lower jaw sheath, edges of both jaw sheaths serrated along their entire length. Labial tooth row formula 2(2)/3(1) in all stages; tooth row A-1 complete; tooth row A-2 interrupted medially, consisting of two pieces of tooth of the same length, the medial gap broadly larger than tooth lines. Posterior tooth rows P-1 slightly longer than P-2, and P-2 longer than P-3 in all stages. P-1 with medial gap, 0.1 mm, 0.3 mm, and 0.1 mm, respectively (in each stage).

After four months preserved in 10% formalin, the tadpoles have a cream background color with brown reticulations on lateral, dorsal, anterior half of the belly, spiracles, tail muscle, and fins. Ventral fin less reticulated than dorsal fin (Fig. 7). The posterior half of the digestive tract is dark brown, iris black (Fig. 7).

**Figure 8.**
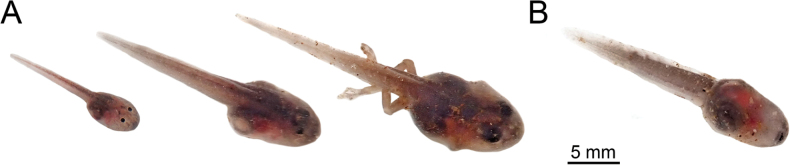
Tadpoles of *Ranitomeyaaquamarina* sp. nov. in life. **A** Dorso-lateral view at Gosner stages 26, 29 and 39 respectively **B** ventral view at Gosner stage 29. Photographs APL.

In life, translucent head, eyes black, anterior portion of body gray in the middle and translucent on sides, posterior body portion gray. Tail musculature uniform gray, dorsal and ventral fins transparent Abdomen mostly transparent, digestive tract gray, heart visible (Fig. 8).

###### Etymology.

The specific epithet ‘*aquamarina*’ is a Latin adjective that means “pale blue-green”, referring to the coloration of the dorsal-lateral stripes of the new species. Another aspect that led us to use this epithet was the metallic blue and greenish tones of the stripes, which resemble seawater. Additionally, aquamarine is a gemstone, which philosophically conveys the value of this discovery.

###### Distribution, habitat, natural history, and conservation.

**Figure 9.**
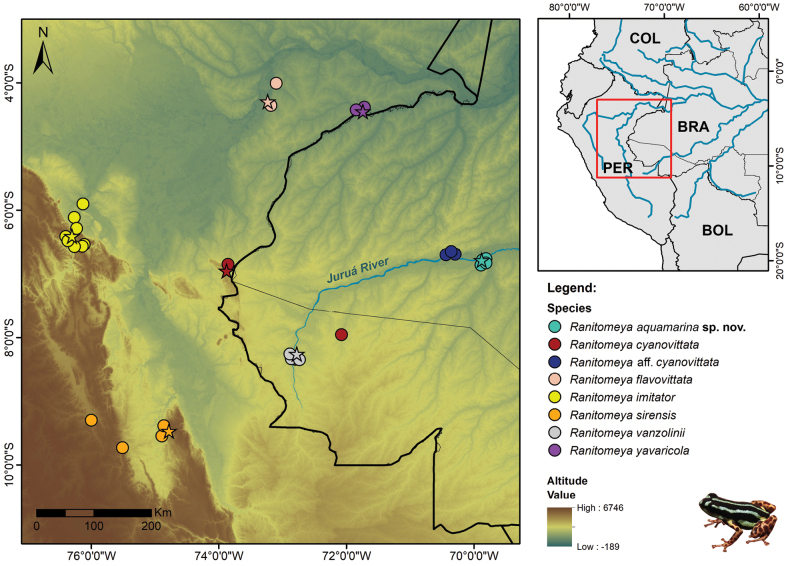
Geographic distribution of the *Ranitomeyaaquamarina* sp. nov. and species of the *R.vanzolinii* clade. Stars indicate type localities of each species. Abbreviations: BRA – Brazil; PER – Peru; COL – Colombia; BOL – Bolivia.

*Ranitomeyaaquamarina* sp. nov. is only known from its type locality, in preserved forests on the Eiru River, a tributary of the Juruá River, near the Comunidade de Santo Antônio, municipality of Eirunepé, state of Amazonas, Brazil (Fig. 9). We sampled four RAPELD modules in the region, and the new species has only been recorded at one site. We did not find the new species living in sympatry with any other species of the genus. However, other Dendrobatoidea occur at the site: *Allobatesfemoralis*, *Allobates* sp. undescribed (A.P. Lima, unpublished data), *Ameeregahahneli* and *A.trivittata*.

**Figure 10.**
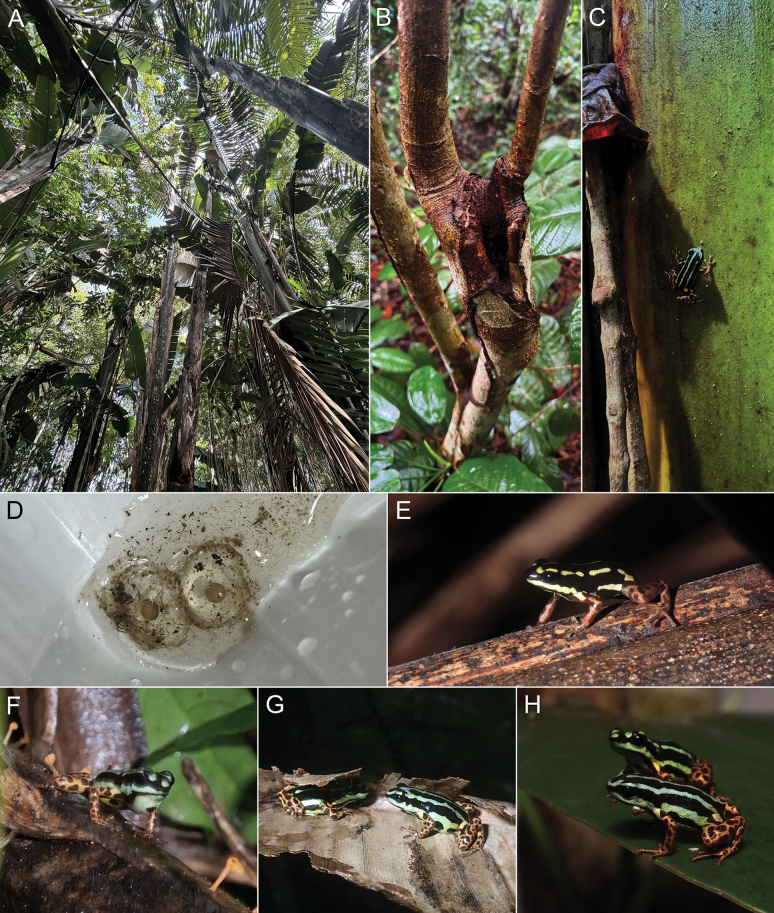
*Ranitomeyaaquamarina* sp. nov. natural history and breeding aspects **A** example of the habitat used by the species **B** phytotelma used by a couple of the species for reproduction **C** adult male climbing (unvouchered) **D** eggs **E** juvenile **F** calling male (INPA-H 47563) **G** couple in cortege (male MPEG 45223 and female MPEG 45222) **H** couple who was in the cited phytotelma (male INPA-H 47568 and female INPA-H 47569). Photographs ATM (**A–E, H**) and APL (**F, G**).

*Ranitomeyaaquamarina* sp. nov. is diurnal, showing greater activity in the early morning and late afternoon. On rainy days, activity lasts throughout the day. Most individuals were observed in clusters of ‘*banananeirabrava*’ (*Phenakospermumguyannense*, Strelitziacaea; Fig. 10A), but the species was also found in a phytotelma in the forest understory, ~ 3 m above the ground (Fig. 10B). Individuals climb vertically through vegetation (Fig. 10C) and are very agile.

Eggs are deposited in water accumulated in cavities in the vegetation. We found eggs (Fig. 10D) and tadpoles in the ‘*banananeirabrava*’ axils and in small holes in trees (Fig. 10B). The eggs are small and brown, wrapped in a thick transparent gelatinous layer. Furthermore, we found tadpoles at different stages of development and metamorphizing in the same area, which suggests that reproduction is prolonged, probably occurring during the entire rain season. Juveniles (*n* = 5; not collected) and adults (*n* = 8) were seen foraging among the dry leaves of the same plants. In juveniles, dorsolateral stripes are uniformly yellow and are not fully formed (Fig. 10E).

Males perform calling perched on vegetation (Fig. 10F). They start calling at dawn (~ 6 am) and remain active until ~ 9 am, with a peak between 7 am and 8 am. After that, their activity remains sporadic until ~ 11 am. They call again in the late afternoon, but with a lower intensity. Most of the time, we observed adults as couples (*n* = 8; Fig. 10G, H), which strongly suggests that the species is monogamous. Males appear to be territorial, responding to and approaching the playback. Additionally, when we captured the females, the respective males called incessantly.

The new species was found in only one of the four sampling sites (5 km RAPELD trails) and appears to be strongly associated with ‘*bananeira brava*’ plants. Therefore, this species is not expected to be abundant nor homogeneously distributed throughout its range. Its known extent of occurrence and area of occupancy are restricted, suggesting that its conservation status deserves attention. Nevertheless, we currently do not have enough information to assign *Ranitomeyaaquamarina* sp. nov. to any IUCN category, and here we classify it as Data Deficient (DD).

## ﻿Discussion

All the species of the *Ranitomeyavanzolinii* species group are distributed across the southwestern Amazonia (Muell et al. 2022; Frost 2025), in the Andean foothills and forests of Peru and Brazil, and most of them are known to have narrow ranges of occurrence (see Fig. 9). The same applies to *Ranitomeyaaquamarina* sp. nov., which is known only from the type locality, on the right bank of the Juruá River. However, the range of the new species is probably greater because, based on its molecular data, we believe that the lineage named as “*Ranitomeya* sp. Envira” (sensu Twomey et al. 2023), occurring ca 60 km to the south, is the same species. Additionally, we suspect that another record 330 km to the north, made through citizen science, corresponds to the new species based on its morphology (photography by Alex Less; iNaturalist 2024). We hope that more sampling in the region will take place soon, as this may provide a better overview of the distribution of the species.

Unlike other dendrobatids (e.g., *Ameerega*, *Dendrobates* and *Phyllobates*), *Ranitomeya* species are especially difficult to detect in fieldwork due to their diminutive body size, conspicuous habits, calls that cannot be heard over long distances, specific microhabitats, and because they are hardly ever seen during the night (Lötters et al. 2007; Brown et al. 2011). These factors contribute to the fact that *Ranitomeya* species are rarely listed in amphibian faunal inventories, even in areas where they occur. In Brazil, when found, the number of individuals observed is very low (e.g., Queiroz et al. 2011; Waldez et al. 2013). This is, for example, the case of *R.defleri*, which has only recently been confirmed in Brazil (Simões et al. 2019), although it was long assumed to be present in the country (Brown et al. 2011). Difficult detection of frogs of the genus *Ranitomeya* in the field resulted in many species being described using fewer than five individuals (e.g., *R.cyanovittata*, *R.defleri*, *R.fantastica*, *R.flavovittata*, *R.sirensis*, and *R.variabilis*).

The genus *Ranitomeya* was recovered to be monophyletic with a posterior probability of 1. Species relationships were found to be mostly consistent with the revisions of Grant et al. (2017) and Brown et al. (2011) but showed some differences from the genomic framework of Muell et al. (2022) and Twomey et al. (2023). This was expected as the first two studies use a multiple-loci approach similar to ours, while the last one uses a genomic approach (Ultraconserved Elements). For example, we recovered the same species groups as defined in Brown et al. (2011): *R.defleri*, *R.variabilis*, *R.reticulata* and *R.vanzolinii* group, which do not correspond to those in Muell et al. (2022). However, we found a low posterior probability between *R.defleri* and *R.toraro* indicating that there are incongruences in the relationships between these species, pointing to the findings of Muell et al. (2022) and Twomey et al. (2023) that their positions need further comparisons. Taken that, some of the interspecies relationships found here should be interpreted with caution. *Ranitomeyaaquamarina* sp. nov. could be confirmed within the *R.flavovittata* clade *sensu*Muell et al. (2022, cit. *Ranitomeya* sp. Envira) but its relationships with its closest relatives, *R.imitator* (here) or *R.flavovittata* and *R.cyanovittata* (Twomey et al. 2023) need to be further evaluated. In addition, our DNA barcoding indicates that some populations (e.g. R.aff.cyanovittata) differentiate at species level.

Our species delimitation results also presented some incongruences to the current *Ranitomeya* taxonomy. While most valid species were correctly recovered as a single OTU, some species were merged within another OTU (*R.flavovittata* and *R.vanzolinii*; *R.benedicta* and *R.fantastica*; *R.ventrimaculata* and *R.reticulata*) and others were split into multiple OTUs (*R.sirensis*, *R.variabilis* and *R.ventrimaculata*). Also, while the new species and others were congruent among all the delimitations, some incongruences were found, specially within the OTUs cited above. The diversity within *R.sirensis*, *R.variabilis* and *R.ventrimaculata* was already discussed before and some nominal taxa (e.g., *R.duellmani*, *R.biolat*, and *R.lamasi*) were already synonymized with the former species. The possible revalidation of *R.biolat* was already discussed in Muell et al. (2022). We also found that the species that clumped in one OTU have low genetic p-distances for 16S (*R.flavovittata* and *R.vanzolinii*, 1,66%; *R.benedicta* and *R.fantastica*, 1.55%; *R.ventrimaculata* and *R.reticulata*, 1.78%). Therefore, we should be cautious when interpreting molecular delimitation analysis, its results are of less significance unless included in an integrative background that includes morphology, ecology, calls, and other evidence that corroborates the results. The systematics and taxonomy of the *Ranitomeya* genus has proven to be complex, with many changes in the species placements and status through time. The use of genomic data helped to better understand its evolution and even stated the possibility of *Ranitomeyaaquamarina* sp. nov. to be recognized as a full species (cit. *Ranitomeya* sp. Envira, Twomey et al. 2023). In any case, an integrative approach is essential for the continuous improvement of taxonomic knowledge within the genus *Ranitomeya*.

The last description of a species of *Ranitomeya* was published more than ten years ago (*R.toraro*; Brown et al. 2011), who presented relevant contribution that filled the knowledge gaps of previously described species, such as calls and tadpoles. However, now advances in integrative taxonomy have the potential to boost the study of *Ranitomeya* diversity. One of the important prerequisites for a thorough investigation of the taxonomy of the frogs of the genus *Ranitomeya* is the use of a wide range of easily comparable predetermined character definitions [for example, methodology of taking morphometric data might follow Randrianiaina et al. (2011) and bioacoustic analyzes should follow standards proposed by Köhler et al. (2017)]. Such an approach would prevent descriptions from being based almost exclusively on color patterns, which are generally highly variable (Brown et al. 2011; Lorioux-Chevalier et al. 2023; Rubio et al. 2024).

Finally, it is generally accepted that the true diversity of frogs is still very poorly known in Amazonia (e.g., Vacher et al. 2020). This fact is doubly true for the region of lower and middle Juruá River. Although we are only taking the first steps to uncover the biodiversity of this area, we already have evidence of the extraordinary richness of the local fauna and we already identified many new candidate species (e.g., Moraes et al. 2022; Lima et al. 2024; Martins et al. 2024). We hope that our research will stimulate more interest in this region, shed more light on its enormous biological wealth and, last but not least, provide important information for its protection.

## Supplementary Material

XML Treatment for Ranitomeya aquamarina

## ﻿Additional information

### Author ORCIDs

Alexander Tamanini Mônico https://orcid.org/0000-0002-2965-8020

Esteban Diego Koch https://orcid.org/0000-0002-1181-403X

Jussara Santos Dayrell https://orcid.org/0000-0003-4211-421X

Jiří Moravec https://orcid.org/0000-0003-4114-7466

Albertina Pimentel Lima https://orcid.org/0000-0003-4586-5633

### Data availability

All of the data that support the findings of this study are available in the main text or Supplementary Information.

The sequences are available at GenBank: PV190292–PV190301 (16S), PV197204– PV197209 (12S), PV189994–PV189997 (COI) and PV191292–PV191297 (cyt-*b*).

The call recordings are available at The Audiovisual Collection of the Museum of Biological Diversity (MDBio), Fonoteca Neotropical Jacques Vielliard (FNJV), UNICAMP (https://www2.ib.unicamp.br/fnjv/):

INPA-H 47561 - FNJV 124331 - https://www2.ib.unicamp.br/fnjv/collection.php?fnjv=124331

INPA-H 47563 - FNJV 124332 - https://www2.ib.unicamp.br/fnjv/collection.php?fnjv=124332

INPA-H 47563 - FNJV 124333 - https://www2.ib.unicamp.br/fnjv/collection.php?fnjv=124333

MPEG 45220 - FNJV 124334 - https://www2.ib.unicamp.br/fnjv/collection.php?fnjv=124334

MPEG 45220 - FNJV 124335 - https://www2.ib.unicamp.br/fnjv/collection.php?fnjv=124335

INPA-H 47566 - FNJV 124336 - https://www2.ib.unicamp.br/fnjv/collection.php?fnjv=124336

INPA-H 47568 - FNJV 124337 - https://www2.ib.unicamp.br/fnjv/collection.php?fnjv=124337

MPEG 45223 - FNJV 124338 - https://www2.ib.unicamp.br/fnjv/collection.php?fnjv=124338

INPA-H 47570 - FNJV 124339 - https://www2.ib.unicamp.br/fnjv/collection.php?fnjv=124339

## Supplementary materials

Supplementary material 1 Morphometric measurements (in mm) of the type specimens of *Ranitomeyaaquamarina* sp. nov. Data type docx https://binary.pensoft.net/file/1316248This dataset is made available under the Open Database License (http://opendatacommons.org/licenses/odbl/1.0/). The Open Database License (ODbL) is a license agreement intended to allow users to freely share, modify, and use this Dataset while maintaining this same freedom for others, provided that the original source and author(s) are credited.Alexander Tamanini Mônico, Esteban Diego Koch, Jussara Santos Dayrell, Jiří Moravec, Albertina Pimentel Lima

Supplementary material 2 Acoustic parameters of advertisement call of *Ranitomeyaaquamarina* sp. nov. Data type docx https://binary.pensoft.net/file/1316249This dataset is made available under the Open Database License (http://opendatacommons.org/licenses/odbl/1.0/). The Open Database License (ODbL) is a license agreement intended to allow users to freely share, modify, and use this Dataset while maintaining this same freedom for others, provided that the original source and author(s) are credited.Alexander Tamanini Mônico, Esteban Diego Koch, Jussara Santos Dayrell, Jiří Moravec, Albertina Pimentel Lima

Supplementary material 3 Species of *Ranitomeya*, *Andinobates*, and *Excidobates* used in phylogenetic analyses, with respective voucher, Genbank acession numbers and references Data type docx https://binary.pensoft.net/file/1316250This dataset is made available under the Open Database License (http://opendatacommons.org/licenses/odbl/1.0/). The Open Database License (ODbL) is a license agreement intended to allow users to freely share, modify, and use this Dataset while maintaining this same freedom for others, provided that the original source and author(s) are credited.Alexander Tamanini Mônico, Esteban Diego Koch, Jussara Santos Dayrell, Jiří Moravec, Albertina Pimentel Lima

Supplementary material 4 Species delimitation results of *Ranitomeya* Data type xlsx https://binary.pensoft.net/file/1316251This dataset is made available under the Open Database License (http://opendatacommons.org/licenses/odbl/1.0/). The Open Database License (ODbL) is a license agreement intended to allow users to freely share, modify, and use this Dataset while maintaining this same freedom for others, provided that the original source and author(s) are credited.Alexander Tamanini Mônico, Esteban Diego Koch, Jussara Santos Dayrell, Jiří Moravec, Albertina Pimentel Lima

Supplementary material 5 Continuation of phylogenetic reconstruction showing the position of *Ranitomeyaaquamarina* sp. nov. Data type tif Explanation note Bayesian inference tree inferred with 16S, 12S, COI, and cyt-*b.* Posterior probability is shown close to nodes (See Fig. 1). https://binary.pensoft.net/file/1316252This dataset is made available under the Open Database License (http://opendatacommons.org/licenses/odbl/1.0/). The Open Database License (ODbL) is a license agreement intended to allow users to freely share, modify, and use this Dataset while maintaining this same freedom for others, provided that the original source and author(s) are credited.Alexander Tamanini Mônico, Esteban Diego Koch, Jussara Santos Dayrell, Jiří Moravec, Albertina Pimentel Lima
