# Supplementary material for: ﻿An Amazonian hidden gem: a new metallic-colored species of Ranitomeya (Anura, Dendrobatidae) from Juruá River basin forests, Amazonas state, Brazil
Source: Zookeys. 2025 Apr 25;1236:51–83. doi: 10.3897/zookeys.1236.146533 (PMC12048821; doi:10.3897/zookeys.1236.146533)
Supplement: Supplementary material 1 — Morphometric measurements (in mm) of the type specimens of Ranitomeyaaquamarina sp. nov. [file zookeys-1236-051_article-146533__-s001.docx]

Table S1. Morphometric measurements (in mm) of adults of the type series of *Ranitomeya aquamarina* sp. nov.

Measurement acronyms are defined in the text. Abbreviations: INPA-H, Instituto Nacional de Pesquisas da Amazônia; MPEG, Museu Paraense Emílio Goeldi; FN, field numbers; M, male; F, female.

*(1/2)*

| **Voucher** | **Sex** | **SVL** | **HL** | **HW** | **IOD** | **UEW** | **MTD** | **TD** | **DET** | **ED** | **SL** | **END** | **BW** | **TSCN** | **IND** | **KK** | **FL** | **TL** | **TaL** | **FoL** | **LT1** | **LT2** | **LT3** | **LT4** | **LT5** |
| --- | --- | --- | --- | --- | --- | --- | --- | --- | --- | --- | --- | --- | --- | --- | --- | --- | --- | --- | --- | --- | --- | --- | --- | --- | --- |
| INPA-H 47561 | M | 16.82 | 4.98 | 5.62 | 2.50 | 1.41 | 0.53 | 0.93 | 0.59 | 2.06 | 1.81 | 1.37 | 5.41 | 0.94 | 1.72 | 13.88 | 6.75 | 6.95 | 4.25 | 6.65 | 1.83 | 3.09 | 5.10 | 6.65 | 4.57 |
| INPA-H 47562 | F | 18.15 | 5.24 | 5.89 | 2.49 | 1.52 | 0.90 | 0.85 | 0.69 | 2.21 | 2.11 | 1.43 | 7.09 | 1.07 | 2.11 | 14.24 | 7.04 | 7.15 | 4.40 | 6.28 | 1.75 | 2.91 | 4.63 | 6.28 | 4.42 |
| INPA-H 47563 | M | 16.92 | 5.04 | 5.70 | 2.42 | 1.39 | 0.69 | 0.83 | 0.61 | 2.13 | 2.00 | 1.41 | 5.72 | 0.95 | 1.94 | 13.62 | 6.76 | 7.05 | 4.35 | 6.67 | 1.57 | 2.85 | 4.78 | 6.67 | 3.89 |
| INPA-H 47564 | M | 17.21 | 5.03 | 5.88 | 2.40 | 1.41 | 0.60 | 0.96 | 0.61 | 1.97 | 1.79 | 1.40 | 5.63 | 1.03 | 2.11 | 14.27 | 6.91 | 7.25 | 4.28 | 7.08 | 1.85 | 3.14 | 5.18 | 7.08 | 4.93 |
| INPA-H 47565 | F | 18.51 | 5.13 | 5.80 | 2.48 | 1.50 | 0.76 | 0.97 | 0.64 | 2.14 | 2.23 | 1.58 | 6.69 | 1.12 | 2.25 | 14.95 | 7.14 | 7.54 | 4.58 | 7.04 | 1.73 | 3.14 | 5.23 | 7.04 | 4.79 |
| INPA-H 47566 | M | 15.38 | 4.84 | 4.88 | 2.36 | 1.23 | 0.64 | 0.87 | 0.65 | 1.83 | 1.72 | 1.32 | 5.05 | 0.81 | 1.63 | 12.88 | 6.64 | 6.25 | 3.62 | 5.97 | 1.48 | 2.81 | 4.40 | 5.97 | 3.86 |
| INPA-H 47568 | M | 17.11 | 5.14 | 5.71 | 2.29 | 1.42 | 0.82 | 0.87 | 0.61 | 1.96 | 2.01 | 1.45 | 5.76 | 0.97 | 2.03 | 13.69 | 6.58 | 7.02 | 3.70 | 7.05 | 1.85 | 2.99 | 4.82 | 7.05 | 4.80 |
| INPA-H 47569 | F | 17.72 | 5.18 | 5.87 | 2.23 | 1.60 | 0.69 | 0.98 | 0.70 | 2.18 | 2.03 | 1.56 | 6.55 | 1.13 | 2.25 | 14.97 | 7.39 | 7.52 | 4.44 | 7.17 | 1.96 | 3.09 | 5.25 | 7.17 | 5.01 |
| INPA-H 47570 | M | 16.73 | 5.27 | 5.54 | 2.21 | 1.59 | 0.61 | 0.94 | 0.68 | 2.04 | 1.91 | 1.40 | 5.59 | 0.97 | 1.98 | 14.03 | 7.22 | 6.97 | 3.70 | 6.48 | 1.76 | 2.94 | 4.84 | 6.48 | 4.39 |
| MPEG 45220 | M | 17.66 | 5.09 | 5.76 | 2.44 | 1.37 | 0.73 | 0.87 | 0.66 | 2.15 | 1.98 | 1.55 | 5.82 | 0.93 | 2.04 | 14.09 | 7.07 | 7.31 | 4.29 | 6.70 | 1.67 | 2.93 | 5.00 | 6.70 | 4.49 |
| MPEG 45221 | F | 17.84 | 5.17 | 5.82 | 2.26 | 1.66 | 0.77 | 1.09 | 0.59 | 2.18 | 1.98 | 1.43 | 6.28 | 1.09 | 2.12 | 14.87 | 7.17 | 7.63 | 4.29 | 6.53 | 1.91 | 3.47 | 5.32 | 6.53 | 5.05 |
| MPEG 45222 | F | 17.32 | 5.13 | 5.68 | 2.27 | 1.66 | 0.73 | 1.01 | 0.70 | 1.98 | 1.96 | 1.49 | 6.18 | 1.09 | 2.05 | 14.47 | 7.32 | 7.48 | 4.14 | 7.03 | 2.00 | 3.02 | 4.90 | 7.03 | 4.92 |
| MPEG 45223 | M | 17.27 | 5.37 | 5.83 | 2.28 | 1.56 | 0.73 | 1.01 | 0.71 | 2.00 | 1.85 | 1.39 | 5.87 | 0.97 | 1.95 | 13.99 | 7.04 | 6.64 | 3.76 | 7.01 | 1.96 | 3.26 | 5.29 | 7.01 | 4.94 |

*(2/2)*

| **Voucher** | **W1TD** | **W1T** | **W2TD** | **W2T** | **W3TD** | **W3T** | **W4TD** | **W4T** | **W5TD** | **W5T** | **AL** | **FAL** | **HaL** | **L1F** | **L2F** | **L3F** | **L4F** | **W1FD** | **W1F** | **W2FD** | **W2D** | **W3FD** | **W3F** | **W4FD** | **W4F** |
| --- | --- | --- | --- | --- | --- | --- | --- | --- | --- | --- | --- | --- | --- | --- | --- | --- | --- | --- | --- | --- | --- | --- | --- | --- | --- |
| INPA-H 47561 | 0.35 | 0.33 | 0.52 | 0.42 | 0.69 | 0.57 | 0.85 | 0.67 | 0.78 | 0.65 | 5.02 | 4.27 | 4.44 | 2.00 | 3.20 | 4.44 | 3.38 | 0.48 | 0.36 | 0.75 | 0.54 | 0.92 | 0.66 | 0.91 | 0.64 |
| INPA-H 47562 | 0.43 | 0.39 | 0.52 | 0.44 | 0.69 | 0.59 | 0.98 | 0.71 | 0.83 | 0.73 | 5.20 | 4.21 | 4.45 | 2.14 | 3.46 | 4.45 | 3.64 | 0.39 | 0.31 | 0.70 | 0.59 | 0.80 | 0.65 | 0.93 | 0.65 |
| INPA-H 47563 | 0.32 | 0.31 | 0.46 | 0.37 | 0.62 | 0.51 | 0.91 | 0.74 | 0.82 | 0.70 | 4.98 | 4.12 | 4.22 | 1.87 | 3.09 | 4.22 | 3.51 | 0.44 | 0.32 | 0.65 | 0.47 | 0.85 | 0.63 | 0.86 | 0.65 |
| INPA-H 47564 | 0.35 | 0.35 | 0.52 | 0.42 | 0.68 | 0.52 | 0.85 | 0.68 | 0.75 | 0.70 | 4.94 | 4.10 | 4.66 | 1.90 | 3.08 | 4.66 | 3.68 | 0.42 | 0.35 | 0.60 | 0.41 | 0.90 | 0.73 | 0.89 | 0.70 |
| INPA-H 47565 | 0.46 | 0.39 | 0.59 | 0.48 | 0.82 | 0.64 | 0.95 | 0.71 | 0.90 | 0.72 | 5.32 | 4.23 | 4.46 | 2.03 | 3.15 | 4.46 | 3.49 | 0.43 | 0.35 | 0.71 | 0.53 | 0.78 | 0.58 | 0.86 | 0.70 |
| INPA-H 47566 | 0.30 | 0.30 | 0.39 | 0.38 | 0.51 | 0.45 | 0.66 | 0.51 | 0.60 | 0.54 | 4.58 | 3.92 | 3.97 | 1.91 | 2.84 | 3.97 | 2.96 | 0.35 | 0.29 | 0.56 | 0.47 | 0.76 | 0.61 | 0.75 | 0.55 |
| INPA-H 47568 | 0.33 | 0.33 | 0.56 | 0.41 | 0.74 | 0.51 | 0.78 | 0.59 | 0.69 | 0.61 | 4.49 | 4.09 | 4.43 | 2.08 | 3.11 | 4.43 | 3.51 | 0.44 | 0.40 | 0.72 | 0.55 | 1.05 | 0.72 | 0.93 | 0.74 |
| INPA-H 47569 | 0.49 | 0.43 | 0.60 | 0.44 | 0.58 | 0.51 | 0.73 | 0.57 | 0.68 | 0.56 | 5.01 | 4.11 | 4.72 | 2.05 | 3.53 | 4.72 | 3.76 | 0.48 | 0.43 | 0.72 | 0.58 | 0.88 | 0.73 | 0.74 | 0.64 |
| INPA-H 47570 | 0.37 | 0.33 | 0.49 | 0.44 | 0.66 | 0.55 | 0.84 | 0.71 | 0.82 | 0.63 | 4.67 | 3.95 | 4.48 | 1.83 | 3.02 | 4.48 | 3.47 | 0.36 | 0.32 | 0.65 | 0.49 | 0.93 | 0.77 | 0.94 | 0.73 |
| MPEG 45220 | 0.42 | 0.38 | 0.57 | 0.44 | 0.76 | 0.54 | 0.93 | 0.70 | 0.84 | 0.59 | 4.41 | 3.99 | 4.65 | 1.97 | 3.30 | 4.65 | 3.46 | 0.42 | 0.40 | 0.65 | 0.51 | 0.94 | 0.68 | 0.83 | 0.61 |
| MPEG 45221 | 0.34 | 0.33 | 0.55 | 0.42 | 0.79 | 0.53 | 1.00 | 0.67 | 0.95 | 0.75 | 5.04 | 4.39 | 4.70 | 2.18 | 3.20 | 4.70 | 3.80 | 0.45 | 0.37 | 1.01 | 0.70 | 1.03 | 0.76 | 0.98 | 0.72 |
| MPEG 45222 | 0.41 | 0.41 | 0.52 | 0.47 | 0.67 | 0.63 | 0.89 | 0.78 | 0.75 | 0.72 | 4.79 | 4.28 | 4.80 | 2.12 | 3.14 | 4.80 | 3.75 | 0.43 | 0.38 | 0.69 | 0.56 | 0.94 | 0.81 | 0.87 | 0.74 |
| MPEG 45223 | 0.41 | 0.41 | 0.55 | 0.47 | 0.71 | 0.62 | 0.84 | 0.73 | 0.84 | 0.72 | 4.88 | 4.07 | 4.71 | 2.05 | 3.52 | 4.71 | 3.78 | 0.55 | 0.46 | 0.78 | 0.68 | 1.05 | 0.84 | 0.93 | 0.73 |
