# Supplementary material for: ﻿An Amazonian hidden gem: a new metallic-colored species of Ranitomeya (Anura, Dendrobatidae) from Juruá River basin forests, Amazonas state, Brazil
Source: Zookeys. 2025 Apr 25;1236:51–83. doi: 10.3897/zookeys.1236.146533 (PMC12048821; doi:10.3897/zookeys.1236.146533)
Supplement: Supplementary material 2 — Acoustic parameters of advertisement call of Ranitomeyaaquamarina sp. nov. [file zookeys-1236-051_article-146533__-s002.docx]

Table S2. Acoustic parameters of advertisement call of *Ranitomeya aquamarina* sp. nov.

Abbreviations: vouchers: INPAH, Instituto Nacional de Pesquisas da Amazônia; MPEG, Museu Paraense Emílio Goeldi; FNJV, Fonoteca Neotropical Jacques Vielliard; AT, air temperature (ºC); NN, number of notes per call; CD, call duration (ms); SBC, silence between calls (s); ND, note duration (ms); SBN, silence between notes (ms); LF, minimum frequency (Hz); HF, maximum frequency (Hz); and DF, dominant frequency (Hz).

| Voucher | | AT | NN | CD | SBC | ND | SBN | LF | HF | DF |
| --- | --- | --- | --- | --- | --- | --- | --- | --- | --- | --- |
| specimen | call recorded |  |  |  |  |  |  |  |  |  |
| INPA-H 47561 | FNJV 124331 | 24.3 | 26 | 842 | 50.5 | 14.8 | 16.3 | 5,077 | 5,693 | 5,426 |
| INPA-H 47561 | FNJV 124331 | 24.3 | 29 | 985 | 27.4 | 14.0 | 22.5 | 4,703 | 5,613 | 5,382 |
| INPA-H 47561 | FNJV 124331 | 24.3 | 21 | 705 | 25.7 | 14.1 | 22.3 | 4,707 | 5,782 | 5,340 |
| INPA-H 47561 | FNJV 124331 | 24.3 | 27 | 937 | - | 14.0 | 21.6 | 4,699 | 5,761 | 5,426 |
| INPA-H 47563 | FNJV 124332 | 25.0 | 32 | 941 | 6.0 | 10.1 | 20.8 | 5,539 | 6,389 | 5,857 |
| INPA-H 47563 | FNJV 124332 | 25.0 | 37 | 1,103 | 8.5 | 9.6 | 19.7 | 5,455 | 6,166 | 5,771 |
| INPA-H 47563 | FNJV 124332 | 25.0 | 38 | 1,151 | 7.7 | 10.9 | 21.6 | 5,247 | 6,139 | 5,685 |
| INPA-H 47563 | FNJV 124333 | 25.0 | 35 | 1,062 | 7.2 | 11.6 | 21.2 | 5,355 | 6,253 | 5,685 |
| INPA-H 47563 | FNJV 124333 | 25.0 | 40 | 1,199 | 31.4 | 10.5 | 20.2 | 5,255 | 6,210 | 5,685 |
| INPA-H 47563 | FNJV 124333 | 25.0 | 33 | 1,064 | - | 10.6 | 22.7 | 4,880 | 6,014 | 5,426 |
| MPEG 45220 | FNJV 124334 | 24.5 | 45 | 1,424 | 10.7 | 13.1 | 19.7 | 5,064 | 6,050 | 5,599 |
| MPEG 45220 | FNJV 124334 | 24.5 | 40 | 1,183 | 12.8 | 13.5 | 18.7 | 5,046 | 6,020 | 5,513 |
| MPEG 45220 | FNJV 124334 | 24.5 | 40 | 1.049 | 8.7 | 11.6 | 18.9 | 4,849 | 6,006 | 5,426 |
| MPEG 45220 | FNJV 124334 | 24.5 | 44 | 1,350 | 14.6 | 12.4 | 18.3 | 5,112 | 6,073 | 5,513 |
| MPEG 45220 | FNJV 124334 | 24.5 | 44 | 1,371 | - | 12.3 | 17.5 | 5,101 | 5,856 | 5,513 |
| MPEG 45220 | FNJV 124334 | 24.5 | 35 | 1,174 | 5.8 | 13.1 | 21.9 | 4,970 | 5,898 | 5,599 |
| MPEG 45220 | FNJV 124334 | 24.5 | 32 | 998 | 115.3 | 11.2 | 21.5 | 5,002 | 5,834 | 5,426 |
| MPEG 45220 | FNJV 124335 | 24.5 | 35 | 1,174 | 16.3 | 14.8 | 20.2 | 5,353 | 5,958 | 5,685 |
| MPEG 45220 | FNJV 124335 | 24.5 | 37 | 1,191 | - | 13.4 | 20.4 | 4,904 | 6,169 | 5,685 |
| INPA-H 47566 | FNJV 124336 | 26.1 | 24 | 762 | 29.1 | 11.9 | 20.4 | 5,740 | 6,553 | 6,288 |
| INPA-H 47566 | FNJV 124336 | 26.1 | 22 | 667 | 13.5 | 11.9 | 18.3 | 5,839 | 6,568 | 6,288 |
| INPA-H 47566 | FNJV 124336 | 26.1 | 24 | 714 | 26.6 | 11.0 | 18.1 | 5,613 | 6,592 | 6,288 |
| INPA-H 47566 | FNJV 124336 | 26.1 | 23 | 676 | 18.1 | 11.6 | 21.2 | 5,269 | 6,600 | 6,288 |
| INPA-H 47566 | FNJV 124336 | 26.1 | 22 | 684 | - | 11.9 | 19.3 | 5,860 | 6,574 | 6,288 |
| INPA-H 47568 | FNJV 124337 | 25.2 | 33 | 938 | 15.7 | 10.3 | 18.9 | 4,893 | 5,957 | 5,599 |
| INPA-H 47568 | FNJV 124337 | 25.2 | 35 | 1,002 | 14.4 | 11.6 | 18.2 | 5,154 | 6,130 | 5,685 |
| INPA-H 47568 | FNJV 124337 | 25.2 | 35 | 1,050 | 8.5 | 11.5 | 20.0 | 5,175 | 6,078 | 5,685 |
| INPA-H 47568 | FNJV 124337 | 25.2 | 35 | 1,068 | 11.0 | 9.7 | 19.3 | 5,265 | 6,056 | 5,685 |
| INPA-H 47568 | FNJV 124337 | 25.2 | 35 | 1,072 | 32.5 | 10.2 | 19.3 | 4,917 | 6,062 | 5,685 |
| INPA-H 47568 | FNJV 124337 | 25.2 | 32 | 990 | 7.9 | 11.8 | 17.2 | 5,255 | 6,035 | 5,599 |
| INPA-H 47568 | FNJV 124337 | 25.2 | 34 | 1,024 | 7.1 | 12.3 | 16.2 | 5,025 | 5,950 | 5,599 |
| INPA-H 47568 | FNJV 124337 | 25.2 | 35 | 970 | 14.7 | 12.3 | 16.7 | 4,982 | 5,927 | 5,513 |
| INPA-H 47568 | FNJV 124337 | 25.2 | 34 | 988 | 12.6 | 12.2 | 17.0 | 5,015 | 5,918 | 5,513 |
| INPA-H 47568 | FNJV 124337 | 25.2 | 34 | 1,105 | 7.4 | 10.6 | 19.0 | 5,048 | 5,875 | 5,513 |
| INPA-H 47568 | FNJV 124337 | 25.2 | 33 | 938 | 7.6 | 11.4 | 18.0 | 5,124 | 5,878 | 5,513 |
| INPA-H 47568 | FNJV 124337 | 25.2 | 34 | 962 | 21.1 | 10.4 | 18.3 | 5,219 | 5,986 | 5,599 |
| INPA-H 47568 | FNJV 124337 | 25.2 | 32 | 957 | - | 10.1 | 20.8 | 5,178 | 6,024 | 5,599 |
| MPEG 45223 | FNJV 124338 | 25.7 | 38 | 1,058 | 10.2 | 11.6 | 17.4 | 4,986 | 6,032 | 5,254 |
| MPEG 45223 | FNJV 124338 | 25.7 | 41 | 1,143 | 5.9 | 10.1 | 18.5 | 5,056 | 6,043 | 5,426 |
| MPEG 45223 | FNJV 124338 | 25.7 | 31 | 858 | 14.4 | 10.1 | 21.2 | 4,949 | 5,927 | 5,426 |
| MPEG 45223 | FNJV 124338 | 25.7 | 37 | 1,030 | - | 11.2 | 15.6 | 4,830 | 5,545 | 4,996 |
| INPA-H 47570 | FNJV 124339 | 25.6 | 21 | 664 | 65.4 | 11.9 | 17.7 | 5,066 | 6,155 | 5,685 |
| INPA-H 47570 | FNJV 124339 | 25.6 | 21 | 658 | 11.7 | 11.7 | 19.6 | 4,990 | 6,024 | 5,685 |
| INPA-H 47570 | FNJV 124339 | 25.6 | 24 | 750 | 11.3 | 11.9 | 18.2 | 5,193 | 6,171 | 5,771 |
| INPA-H 47570 | FNJV 124339 | 25.6 | 21 | 647 | - | 12.3 | 21.3 | 4,965 | 6,101 | 5,685 |
|  |  |  |  |  |  |  |  |  |  |  |
|  |  |  |  |  |  |  |  |  |  |  |
|  |  |  |  |  |  |  |  |  |  |  |
|  |  |  |  |  |  |  |  |  |  |  |
