# Supplementary material for: ﻿An Amazonian hidden gem: a new metallic-colored species of Ranitomeya (Anura, Dendrobatidae) from Juruá River basin forests, Amazonas state, Brazil
Source: Zookeys. 2025 Apr 25;1236:51–83. doi: 10.3897/zookeys.1236.146533 (PMC12048821; doi:10.3897/zookeys.1236.146533)
Supplement: Supplementary material 3 — Species of Ranitomeya, Andinobates, and Excidobates used in phylogenetic analyses, with respective voucher, Genbank acession numbers and references [file zookeys-1236-051_article-146533__-s003.docx]

**Table S3.** Species of *Ranitomeya*, *Andinobates* and *Excidobates* used in phylogenetic analyses, with respective voucher, Genbank acession number and references.

| **Species** | **Voucher** | **Genbank accession number** | | | | **Reference** |
| --- | --- | --- | --- | --- | --- | --- |
|  |  | **12S** | **16S** | **COI** | **CYTB** |  |
| *Andinobates claudiae* | Unvouchered | - | DQ371315 | - | DQ371334 | Roberts et al. (2006) |
| *A. minutus* | Unvouchered | - | AF128589 | - | AF128591 | unpublished |
| *Excidobates captivus* | Unvouchered | - | EU325900 | - | EU325902 | Twomey and Brown (2008) |
| *E. misteriosus* | Unvouchered | - | DQ371314 | - | DQ371333 | Roberts et al. (2006) |
| *R.* aff. *cyanovittata* | APL24650 | [Processing] | [Processing] | [Processing] | [Processing] | A.P. Lima (pers. data) |
| *R.* aff. *cyanovittata* | APL24702 | - | [Processing] | - | - | A.P. Lima (pers. data) |
| *R.* aff. *cyanovittata* | APL24703 | - | [Processing] | - | - | A.P. Lima (pers. data) |
| *R.* aff. *cyanovittata* | APL24704 | - | [Processing] | - | - | A.P. Lima (pers. data) |
| *R.* aff. *cyanovittata* | APL24732 | [Processing] | [Processing] | [Processing] | [Processing] | A.P. Lima (pers. data) |
| *R.* aff. *cyanovittata* | APL24733 | [Processing] | [Processing] | - | [Processing] | A.P. Lima (pers. data) |
| *R.* aff. *cyanovittata* | APL24735 | - | [Processing] | - | - | A.P. Lima (pers. data) |
| *R.* aff. *cyanovittata* | APL24736 | - | [Processing] | - | - | A.P. Lima (pers. data) |
| *R.* aff. *cyanovittata* | APL24738 | - | [Processing] | - | - | A.P. Lima (pers. data) |
| *R.* aff. *cyanovittata* | APL24826 | [Processing] | [Processing] | [Processing] | [Processing] | A.P. Lima (pers. data) |
| *R.* aff. *cyanovittata* | APL24845 | [Processing] | [Processing] | - | [Processing] | A.P. Lima (pers. data) |
| *R.* aff. *cyanovittata* | APL24846 | [Processing] | [Processing] | [Processing] | [Processing] | A.P. Lima (pers. data) |
| *R. aquamarina* **sp. nov.** | INPA-H 47561 | [Processing] | [Processing] | [Processing] | [Processing] | This work |
| *R. aquamarina* **sp. nov.** | INPA-H 47562 | - | [Processing] | - | - | This work |
| *R. aquamarina* **sp. nov.** | INPA-H 47563 | - | [Processing] | - | - | This work |
| *R. aquamarina* **sp. nov.** | INPA-H 47564 | - | [Processing] | - | - | This work |
| *R. aquamarina* **sp. nov.** | INPA-H 47565 | [Processing] | [Processing] | - | [Processing] | This work |
| *R. aquamarina* **sp. nov.** | INPA-H 47568 | [Processing] | [Processing] | [Processing] | [Processing] | This work |
| *R. aquamarina* **sp. nov.** | INPA-H 47570 | [Processing] | [Processing] | [Processing] | [Processing] | This work |
| *R. aquamarina* **sp. nov.** | MPEG 45220 | - | [Processing] | - | - | This work |
| *R. aquamarina* **sp. nov.** | MPEG 45221 | [Processing] | [Processing] | - | [Processing] | This work |
| *R. aquamarina* **sp. nov.** | MPEG 45223 | [Processing] | [Processing] | [Processing] | [Processing] | This work |
| *R. amazonica* | JLB07_10 | - | JN651272 | - | JN635959 | Brown et al. (2011) |
| *R. amazonica* | JLB07_17 | - | JN651251 | - | JN635940 | Brown et al. (2011) |
| *R. amazonica* | JLB08_014 | - | JN635839 | - | JN635912 | Brown et al. (2011) |
| *R. amazonica* | JLB08_015 | - | JN635840 | - | JN635913 | Brown et al. (2011) |
| *R. benedicta* | Shucush JLB07 | - | EU736219 | - | - | Brown et al. (2011) |
| *R. benedicta* | Shucush JLB07 2 | - | EU736221 | - | - | Brown et al. (2011) |
| *R. cyanovittata* | MCP10263 | MF624240 | MF624240 | MF614328 | MF614231 | Grant et al. (2017) |
| *R. cyanovittata* | MCP10265 | MF624241 | MF624241 | MF614329 | MF614232 | Grant et al. (2017) |
| *R. cyanovittata* | 186 | - | HM038422 | - | HM038425 | Perez-Peña et al. (2010) |
| *R. defleri* | JLB08_004 | - | GU062191 | - | GU062192 | Twomey et al. (2009) |
| *R. defleri* | JLB08_005 | - | JN635833 | - | JN635906 | Brown et al. (2011) |
| *R. defleri* | JLB08_006 | - | JN635834 | - | JN635907 | Brown et al. (2011) |
| *R. defleri* | JLB08_008 | - | JN635835 | - | JN635908 | Brown et al. (2011) |
| *R. fantastica* | JLB_07_041 | - | EU736209 | - | EU736181 | Brown et al. (2011) |
| *R. fantastica* | DfHCQ2 | - | AF412469 | - | AF412497 | Symula et al. (2001) |
| *R. fantastica* | Huallaga JLB07 | - | EU736215 | - | EU736185 | Brown et al. (2011) |
| *R.* aff. *fantastica* | DfTY26a | - | AF412474 | - | AF412502 | Symula et al. (2001) |
| *R.* aff. *fantastica* | PE PMans 017 2 R | - | JN635848 | - | JN635921 | Brown et al. (2011) |
| *R.* aff. *fantastica* | Varadero JLB07 | - | EU736208 | - | EU736180 | Brown et al. (2011) |
| *R.* aff. *fantastica* | Conveto JLB07 2 | - | EU736214 | - | EU736184 | Brown et al. (2011) |
| *R.* aff*. flavovittata* | 344 Tahauyo 16 | - | JN635862 | - | JN635930 | Brown et al. (2011) |
| *R.* aff*. flavovittata* | 42 2 Tahauyo03 | - | JN635858 | - | JN635928 | Brown et al. (2011) |
| *R.* aff*. flavovittata* | Tahauyo Tahu JB 2 | - | JN635860 | - | JN635929 | Brown et al. (2011) |
| *R.* aff*. flavovittata* | UN | - | DQ371317 | - | DQ371336 | Roberts et al. (2006) |
| *R. imitator* | aRanImi1 | CM064416 | CM064416 | - | CM064416 | Unpublished |
| *R. imitator* | DiHCOS | - | AF412486 | - | AF412514 | Symula et al. (2001) |
| *R. imitator* | DiHCQ1a | - | AF412480 | - | AF412508 | Symula et al. (2001) |
| *R. imitator* | DiHCQ1b | - | AF412476 | - | AF412504 | Symula et al. (2001) |
| *R. imitator* | DiHCQ1c | - | AF412477 | - | AF412505 | Symula et al. (2001) |
| *R. imitator* | DiNcha | - | AF412478 | - | AF412506 | Symula et al. (2001) |
| *R. imitator* | DiNPon | - | AF412479 | - | AF412507 | Symula et al. (2001) |
| *R. imitator* | DiNYur1 | - | AF412481 | - | AF412509 | Symula et al. (2001) |
| *R. imitator* | DiNYur2 | - | AF412482 | - | AF412510 | Symula et al. (2001) |
| *R. imitator* | DiSeda1 | - | AF412483 | - | AF412511 | Symula et al. (2001) |
| *R. imitator* | DiSeda2 | - | AF412484 | - | AF412512 | Symula et al. (2001) |
| *R. imitator* | DiShap | - | AF412485 | - | AF412513 | Symula et al. (2001) |
| *R. imitator* | DiTY26a | - | AF412488 | - | AF412516 | Symula et al. (2001) |
| *R. imitator* | DiTY26b | - | AF412489 | - | AF412517 | Symula et al. (2001) |
| *R. imitator* | DiAchi2 | - | AF412490 | - | AF412518 | Symula et al. (2001) |
| *R. imitator* | DiHCOS | - | AF412487 | - | AF412515 | Symula et al. (2001) |
| *R. reticulata* | 133 112 Q Qblanco | - | JN635810 | - | - | Brown et al. (2011) |
| *R. reticulata* | TNHC61143 | AY326029 | AY326029 | - | - | Darst and Cannatella (2004) |
| *R. reticulata* | 3155 | DQ502119 | DQ502119 | DQ502827 | DQ502551 | Grant et al. (2006) |
| *R. reticulata* | 528 | - | AF412467 | - | AF412495 | Symula et al. (2001) |
| *R. reticulata* | Dret | EU342686 | EU342686 | - | - | Santos et al. (2009) |
| *R. reticulata* | DretAchi1 | - | AF482786 | - | AF482801 | Symula et al. (2001) |
| *R. reticulata* | TNHC61143 | AY364567 | AY364567 | - | - | Santos et al. (2003) |
| *R.* aff. *sirensis* 2 | 163 Dlamasi | JN635776 | JN635830 | - | JN635903 | Brown et al. (2011) |
| *R.* aff. *sirensis* 2 | JCS40A | HQ290986 | HQ290986 | - | HQ290923 | Santos and Cannatella (2011) |
| *R.* aff. *sirensis* 2 | JLB00202 Codo | - | JN635856 | - | JN635925 | Brown et al. (2011) |
| *R.* aff. *sirensis* 2 | JLB08 098 2 | - | JN635847 | - | JN635919 | Brown et al. (2011) |
| *R.* aff. *sirensis* 2 | 5b1_17 | - | JN635854 | - | JN635924 | Brown et al. (2011) |
| *R. sirensis* | JLB07032 | - | JN635825 | - | - | Brown et al. (2011) |
| *R. sirensis* | JLB07031 | - | JN635826 | - | - | Brown et al. (2011) |
| *R.* aff. *sirensis* 1 | 348_10 | - | JN635870 | - | JN635932 | Brown et al. (2011) |
| *R.* aff. *sirensis* 1 | 350 s8 3b 1 | - | JN635874 | - | JN635933 | Brown et al. (2011) |
| *R. summersi* | Chipaota JLB07 | - | EU736217 | - | EU736187 | Brown et al. (2011) |
| *R. summersi* | JLB08_72 | - | JN635844 | - | JN635917 | Brown et al. (2011) |
| *R. summersi* | Sauce JLB07 | - | EU736218 | - | EU736188 | Brown et al. (2011) |
| *R. toraro* | OMNH37440 | DQ502232 | DQ502232 | DQ502905 | DQ502665 | Grant et al. (2006) |
| *R. toraro* | LSUMZ16969 | - | EU342676 | - | - | Santos et al. (2009) |
| *R. uakarii* | Manati_25_1_15 | - | JN635813 | - | JN635892 | Brown et al. (2011) |
| *R. uakarii* | SS | - | DQ371316 | - | DQ371335 | Roberts et al. (2006) |
| *R. uakarii* | THY11 6 | - | JN635812 | - | JN635891 | Brown et al. (2011) |
| *R. vanzolinii* | SS | - | AF128598 | - | AF128600 | Unpublished |
| *R. vanzolinii* | OMNH36036 | DQ502236 | DQ502236 | DQ502908 | DQ502669 | Grant et al. (2006) |
| *R. vanzolinii* | OMNH36035 | DQ502067 | DQ502067 | DQ502498 | DQ502789 | Grant et al. (2006) |
| *R. vanzolinii* | OMNH36037 | DQ502068 | DQ502068 | DQ502790 | DQ502499 | Grant et al. (2006) |
| *R. variabilis* | OMNH34091 | DQ502069 | DQ502069 | DQ502791 | DQ502500 | Grant et al. (2006) |
| *R. variabilis* | 42PE Nuata JLB07 | - | JN651250 | - | JN635939 | Brown et al. (2011) |
| *R. variabilis* | Tahuayo 882004 | - | JN635821 | - | JN635899 | Brown et al. (2011) |
| *R. variabilis* | 49PE Bonilla 2004 | - | JN651257 | - | JN635946 | Brown et al. (2011) |
| *R. variabilis* | JLB08_004 | - | JN635832 | - | JN635905 | Brown et al. (2011) |
| *R. variabilis* | JLB08_011 | - | JN635837 | - | JN635910 | Brown et al. (2011) |
| *R. variabilis* | QCAZ16566 | AY364570 | AY364570 | - | - | Brown et al. (2011) |
| *R. variabilis* | Shuc. JLB07 | - | JN651248 | - | JN635937 | Brown et al. (2011) |
| *R. variabilis* | KS27 | EU342680 | EU342680 | - | JN635884 | Santos et al. (2009) |
| *R. variabilis* | 5a 1A 1611 19 | - | JN635820 | - | JN635898 | Brown et al. (2011) |
| *R.* aff. *variabilis* | Saposoa 04 1 4 | - | JN635818 | - | JN635896 | Brown et al. (2011) |
| *R.* aff. *variabilis* | Saposoa 05 02 14 | - | JN635819 | - | JN635897 | Brown et al. (2011) |
| *R.* aff. *variabilis* | JLB06 0001 | - | JN651266 | - | JN635953 | Brown et al. (2011) |
| *R.* aff. *variabilis* | JLB06 012 | - | JN651265 | - | JN635952 | Brown et al. (2011) |
| *R. ventrimaculata* | 161 Callegarii | - | JN635827 | - | JN635901 | Brown et al. (2011) |
| *R. ventrimaculata* | AY263246 | - | AY263246 | - | - | Vences et al. (2003) |
| *R. ventrimaculata* | QCAZ16559 | HQ290979 | HQ290979 | - | HQ290556 | Santos and Cannatella (2011) |
| *R. ventrimaculata* | QCAZ16561 | AY364566 | AY364566 | - | - | Santos et al. (2003) |
| *R. ventrimaculata* | QCAZ16560 | EU342688 | EU342688 | - | JN635887 | Santos et al. (2009) |
| *R.* aff. *ventrimaculata* 1 | 105mc | - | EU201084 | - | - | Fouquet et al. (2007) |
| *R.* aff. *ventrimaculata* 1 | TNHC64418 | EU342681 | EU342681 | - | - | Santos et al. (2009) |
| *R.* aff. *ventrimaculata* 2 | 75BM | - | EU201083 | - | - | Fouquet et al. (2007) |
| *R.* aff. *ventrimaculata* 2 | BPN664 | - | DQ163075 | - | DQ163066 | Noonan and Wray (2006) |
| *R.* aff. *ventrimaculata* 2 | BPN744 | - | DQ163080 | - | DQ163069 | Noonan and Wray (2006) |
| *R. yavaricola* | SS_180 | - | HM038423 | - | HM038427 | Perez-Peña et al. (2010) |
| *R. yavaricola* | SS_181 | - | HM038424 | - | HM038428 | Perez-Peña et al. (2010) |
