# Supplementary figures and images for: ﻿An Amazonian hidden gem: a new metallic-colored species of Ranitomeya (Anura, Dendrobatidae) from Juruá River basin forests, Amazonas state, Brazil
Source: Zookeys. 2025 Apr 25;1236:51–83. doi: 10.3897/zookeys.1236.146533 (PMC12048821; doi:10.3897/zookeys.1236.146533)

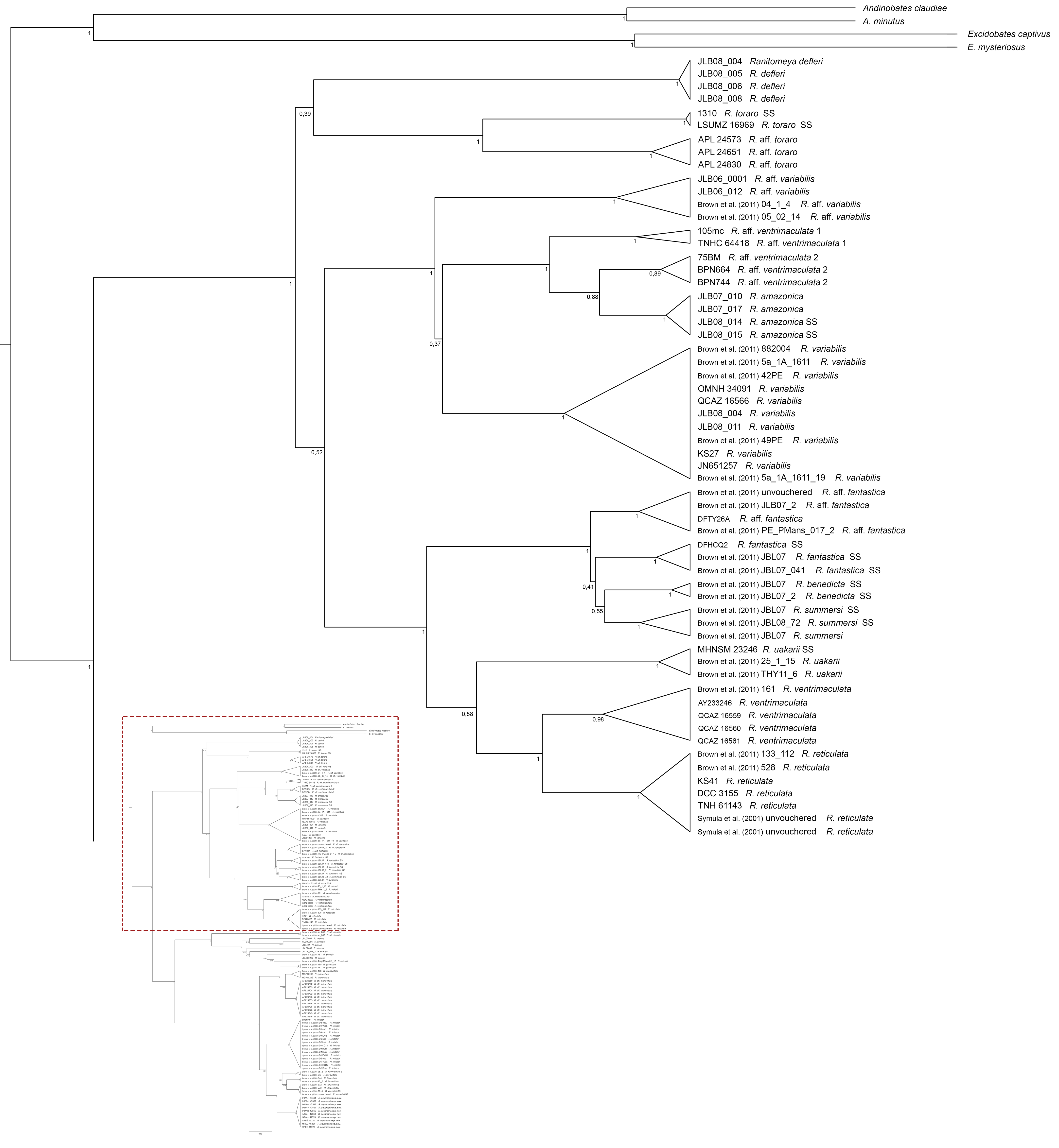

Supplement: Supplementary material 5 — Continuation of phylogenetic reconstruction showing the position of Ranitomeyaaquamarina sp. nov. [file zookeys-1236-051_article-146533__-s005.tif]
